# Influence of phylogenetic structure and climate gradients on geographical variation in the morphology of Mexican flycatcher forests assemblages (Aves: Tyrannidae)

**DOI:** 10.7717/peerj.6754

**Published:** 2019-10-15

**Authors:** Gala Cortés-Ramírez, César A. Ríos-Muñoz, Adolfo G. Navarro-Sigüenza

**Affiliations:** 1 Museo de Zoología “Alfonso L. Herrera”, Facultad de Ciencias, Universidad Nacional Autónoma de México, Mexico City, Mexico; 2 Posgrado en Ciencias Biológicas, Universidad Nacional Autónoma de México, Mexico City, Mexico; 3 Laboratorio de Arqueozoología, Instituto Nacional de Antropología e Historia, Mexico City, Mexico

**Keywords:** Ecomorphology, Phylogenetic structure, Tyrannidae, Morphological variation, Climatic gradients

## Abstract

Morphological variation is strongly related to variation in the ecological characteristics and evolutionary history of each taxon. To explore how geographical variation in morphology is related to different climatic gradients and phylogenetic structure, we analyzed the variation of morphological traits (body size, bill, and wing) of 64 species of tyrant flycatchers (Tyrannidae) distributed in Mexico. We measured these morphological traits in specimens from biological collections and related them to the climatic and topographic data of each collection locality. We performed the analyses separately at two levels: (1) the regional level and (2) the assemblage level, which was split into (assemblage I) lowland forests and (assemblage II) highland forests and other vegetation types. We also calculated the phylogenetic structure of flycatchers of each locality in order to explore the influence of climatic variables and the phylogenetic structure on the morphological variation of tyrant flycatchers, by means of linear mixed-effects models. We mapped the spatial variation of the relationship between morphological traits and environmental gradients, taking into account the phylogenetic structure. Important climatic variables explaining the morphological variation were those of temperature ranges (seasonality) and the results suggest that the phylogenetic clustering increases towards the highlands of Sierra Madre Oriental and Sierra Madre del Sur, and the lowlands of Balsas Depression. For the regional level, the spatial distribution of body size showed a pattern coincident with Bergmann’s rule, with increasing in size from south to north. In the tropical lowland forests assemblage, body size tend to increase in seasonally dry forests (western Mexico) and decrease in the humid ones (eastern Mexico). In the assemblage of highland forests and other types of vegetation, morphological trait values increased northeast to southwest. Phylogenetic structure helped to explain the variation of morphology at the assemblage level but not at the regional level. The patterns of trait variation in the lowland and highland assemblages suggest that parts of morphological variation are explained both by the climatic gradients and by the lineage relatedness of communities. Overall, our results suggest that morphological variation is best explained by a varied set of variables, and that regression models representing this variation, as well as integrating phylogenetic patterns at different community levels, provide a new understanding of the mechanisms underlying the links among biodiversity, its geographical setting, and environmental change.

## Introduction

A long-standing goal in ecology and evolutionary biology is to understand the relationships among morphological diversity, evolutionary history, environment and geographic distribution. Environmental drivers of morphological diversity across geography have been extensively studied in many regions with different taxonomic groups, at different geographic, taxonomic and functional scales (Losos & Miles, 1994; Cavender-Bares et al., 2009; Kluge & Kessler, 2011; Violle et al., 2014; Jarzyna et al., 2015; Jarzyna & Jetz, 2016; Lawing et al., 2017; Schneider et al., 2017; Seeholzer, Claramunt & Brumfield, 2017; Phillips et al., 2018; Mazel et al., 2018). As a result of previous studies that analyze the role of environment and geography as promoters of morphological diversity, patterns of gradual variation of traits have been detected for many groups. Climate seems to be one of the main environmental promoters of morphological variation, strongly influencing the variation of morphological traits across species and regions (e.g., James, 1970; Graves, 1991; Kivelä et al., 2011; Maestri et al., 2016; Xu et al., 2017). However, the role of climate and other environmental variables is poorly understood. Even though many studies have demonstrated its associations with morphological traits, the question remains to what extent and by which mechanisms such associations are maintained and may influence distribution patterns (Violle et al., 2014). It has been suggested that several variables may act simultaneously, promoting morphological variation at many taxonomic and geographic scales.

Morphological diversity across species is driven by several ecological and evolutionary processes and is usually studied as the evolution of form and function, or ecomorphology (Losos & Miles, 1994; Ricklefs, 2012; Dehling et al., 2014; Seeholzer, Claramunt & Brumfield, 2017; Phillips et al., 2018). Also, variation in morphological diversity within communities can have effects in structuring broad-scale biogeographical patterns of species richness along climatic and geographical gradients (Deutsch et al., 2008; Cicero & Koo, 2012). Morphological variation is related to ecology and reflects a response to biotic and abiotic environmental factors, and it may determine species’ responses to climate change (Wainwright & Reilly, 1994; Pontarotti, 2010; Cicero & Koo, 2012). Climatic variables, such as temperature and precipitation, are recognized as major factors determining geographical patterns of morphological variation (Hawkins et al., 2007). For instance, bill size increases with higher temperatures, supporting the hypothesis that larger bills are an adaptation to release heat while minimizing evaporative water loss in hot, dry environments (Greenberg et al., 2012). In this way, overall bill size may be related to physiological responses to regional climates, and the season of critical thermal stress may vary geographically, even on relatively small spatial scales (Campbell-Tennant et al., 2015; Danner & Greenberg, 2015).

Other factors such as evolutionary history also have been found to determine geographical gradients in species variation (Jetz & Rahbek, 2002; Kissling, 2007). For instance, habitat filtering is an ecological process by which species are eliminated from a community because of morphological or ecological similarity with other established members of the community (Wainwright & Reilly, 1994). Under this interpretation, the variation of morphological variables across communities and geography is proportional to the amount of phylogenetic dissimilarity among communities (Pillar & Duarte, 2010), taking into account that morphology is structured by phylogeny at the species level if there is phylogenetic signal. Morphological variation occurs within and across species, so the complex interaction of evolutionary history and environment makes it difficult to identify the underlying causes of broad scale patterns of variation (Endler, 1977; Ricklefs & Miles, 1994; Violle et al., 2014; Forister et al., 2015).

The recognition of the promoters of broad scale patterns of morphological variation is challenging due to the differential response of organisms’ traits to environmental variation and geographical settings (Violle et al., 2014). This limits our ability to elucidate the causes and consequences of the patterns of species’ morphological diversity. For instance, the geographical patterns of community structure and morphological variation in response to climatic gradients has shown contrasting effects of the same environmental variables (e.g., Forister et al., 2015; Pol et al., 2016; Lawing et al., 2017). To understand how morphological diversity arises, it is necessary to explore and quantify how species’ morphological traits are related to their ecology, how they vary geographically along environmental gradients, consider both large and small spatial scales in the same region, and account for the historical contingencies limiting the distribution of species assemblages and their traits (Cavender-Bares et al., 2009). In this sense, phylogenetic structure and distributional data provide the historical framework to quantify ecological, geographical and evolutionary patterns, in order to infer the processes that established them (Saito et al., 2016; Sobral & Cianciaruso, 2016; Phillips et al., 2018). Also, quantifying the geographical distribution of morphological variation may help disentangle trade-offs found in the relationship between morphology and environmental and phylogenetic variables. Then, analyses of the distribution of morphological variation are necessary for improving regional and global predictions of morphological and functional change (Diniz-Filho, 2004; Rodríguez & Ojeda, 2014).

To evaluate broad scale patterns of morphological variation and the underlying processes which promote them, it is necessary to quantify the distribution of morphological traits in relation to the ecology of related functional groups of species. In that sense, some authors have found that the global patterns of functional richness are associated with environmental variables (Kissling, Böhning-Gaese & Jetz, 2009; Brum et al., 2012). To describe how morphology varies geographically with environment, we explored the spatial distribution of a set of morphological variables in relation to climatic gradients of the assemblages of species present in Mexico of a mainly insectivorous monophyletic clade of birds, the tyrant flycatchers (Tyrannidae, sensu (Tello et al., 2009)). This taxon also belongs to a functional group of bird species that use insects and arthropods as their main food resource (Hespenheide, 1971; Sherry, 1984). The family includes more than 400 species distributed across the Americas (IOU, 2018) occurring in almost every habitat. They are adapted to different elevations and occupy all vertical forest strata (Fitzpatrick, 2004; Ridgely & Tudor, 2009). We chose the Tyrannidae of Mexico as a model system because: (1) they are widely distributed in the country (Ridgely et al., 2005; Berlanga et al., 2008); (2) the natural history, phylogenetic structure, and functional significance of their morphological traits is relatively well known (Ohlson, Fjeldså& Ericson, 2008; Tello et al., 2009); (3) their morphology can be related to their ecology (e.g., Fitzpatrick, 1980; Fitzpatrick, 1981; Fitzpatrick, 1985); and (4) their morphology varies across environmental and geographical gradients (Brum et al., 2012).

Our main goal was to investigate the variation of morphology across geography and to determine the relationship of environmental climatic gradients as explanatory factors of morphological function-related traits. We have considered the phylogenetic structure of Mexican flycatchers as a factor that may help to explain how broad scale patterns in species variation are established and how historical contingencies influence the response of morphological variation to the environment. Our specific objectives were to test (1) whether climate conditions (temperature, precipitation, and their seasonality), are associated with the observed variation in morphology across tyrant flycatchers assemblages; (2) the influence of the phylogenetic structure of assemblages on the geographic distribution of morphological variation and its response to climate; and (3) to map the spatial distribution of morphological variation along climatic gradients. Because traits are related to the ecology of the organism, for instance foraging behavior or habitat use (Fitzpatrick, 1985), morphological variation is expected to reflect species’ responses to environmental gradients. Then, the approach we used takes into account varied ranges in climate and seasonality within a lineage, abiotic variables influencing the geographic distribution of species, and the phylogenetic relationships among the tyrant flycatchers. Taking into account phylogenetic relationships within a community by accounting for phylogenetic structuring may help to understand the influence of the composition of a community on the response of traits to environmental variation (Bonetti & Wiens, 2014; Maestri et al., 2016).

### Hypothesis and assumptions

Given that climatic gradients and phylogenetic structure of an area potentially play a role as promoters or constrainers of morphological variation, and because this role may vary in strength and direction, we analyzed the morphological data by constructing regression models in order to explain the relationship between morphology, environment and phylogenetic structure. We hypothesized that, once historical and geographic factors are accounted for: Hypothesis (1) climate gradients explain morphological change across geography; and hypothesis (2) phylogenetic structure of a community influence morphological variation of the co-occurring species. To support hypothesis 1, morphology will show clinal variation related to one or more climatic variables, and a latitudinal pattern when the model is translated into a map. Conversely, latitudinal variation in morphology is likely to be affected by the phylogenetic composition of the area, that is, the variation of morphological traits across geography is expected to be proportional to the amount of phylogenetic dissimilarity among communities (Duarte, 2011). Phylogenetically clustered areas are expected to show different patterns of morphological variation than areas that are phylogenetically overdispersed. Because of the tendency of species to remain in an environmental space similar to that of their ancestors (Wiens & Graham, 2005) we expect that morphological variation within assemblages will be constrained. Phylogenetically clustered assemblages are more likely to be restricted in their climatic ranges, whereas phylogenetically overdispersed assemblages are more likely to be found in the transition zones where there is a high species turnover (Graham et al., 2009). To support hypothesis 2, we would expect that morphological change cannot solely be explained by climatic variables, but that phylogenetic structure is also significantly associated to variation in morphology. Phylogenetic structure alone is also unlikely to explain the variation of morphology; instead it is expected to influence morphology along with climatic variables, meaning that the response of the trait could be driven by either environmental filtering (species are filtered from a community due to morphological or ecological similarity with other co-occurring species), other biotic interactions (e.g., competition), or random factors (Cavender-Bares et al., 2009; Lawing et al., 2017).

## Methods

### Morphological traits data and data treatment

#### Morphological data

In order to construct regression models of environmentally-related morphological variation, the morphological traits were associated to locality-specific climate, topographic and phylogenetic structure data. We obtained morphological data from a sample of 296 skin specimens from 60 species of Tyrannidae distributed in Mexico (Table S1). We measured five traits (Claramunt, 2010, following recommendations by Eck et al., 2011): body size (using mass data as a proxy), bill length, bill width, and bill depth (the last two taken at the anterior border of the nostrils), and wing chord (wing length from the carpal joint to the tip of the longest primary feather without flattening the wing). We selected these traits because they have been associated use of environmental space in birds (Miles & Ricklefs, 1984). Size is a significant attribute at all levels of organization, as it predicts and explains the variation of many organismal and species traits, from the proportion of parts to metabolic rates to the distribution patterns (Schmidt-Nielsen, 1984; Brown, 1995; Diniz-Filho, 2004; Bonner, 2011). Bill size can be positively correlated with temperature in avian taxa (Allen’s rule), and the common explanation for this pattern is that larger surface area of the appendage functions to dissipate excess heat in warm climates and small area to retain heat in cold climates (Symonds & Tattersall, 2010; Greenberg et al., 2012). The bill is also the functional trait by which birds obtain food, so it can be related to habitat and ecomorphological variation (Mazer & Wheelwright, 1993; Jones, Purvis & Quicke, 2012). The relative variation of bill measures represents its variation in size and shape. Finally, wing chord plays a role in determining the aerodynamics and mechanical aspects of the avian wing, thus it interacts with the effective exploitation of habitat; so it is strongly related with ecology and behavior (Hamilton, 1961; Lockwood, Swaddle & Rayner, 1998; Swaddle & Lockwood, 1998; Gatesy & Dial, 1996). Together, body size, bill size and wing chord represent morphological traits that are related to the flycatcher ecology.

In general, we only measured adult male specimens to homogenize the data set and to avoid morphological variations associated with sexual dimorphism. In some cases, we had to measure female specimens (∼8% specimens) to complete the sample, and used these data based on a previous test (Cortés-Ramírez, Ríos-Muñoz & Navarro-Sigüenza, 2012) that showed that the variation between sexes is smaller than interspecific variation (*sensu* Claramunt, 2010). We took all the measurements with digital or analog Mitutoyo calipers, with a precision of 0.01 mm. For statistical analysis we used *natural log*-transformed measures in order to normalize the dataset, and because all morphological measurements may scale with overall body size, we made bill and wing size measurements relative to body size by dividing each measurement by body mass. Relative variation of the three bill measurements was obtained by performing a principal component analysis (PCA) to reduce the dimensionality of bill variation (Table S2), retaining the first principal component as representative of bill variation and size. The first principal component represented 86% of bill variation and overall size of the bill. Each morphological variable was evaluated independently from the other variables.

### Environmental and geographic data

#### Climatic variables

We considered the geographic location of each specimen to obtain locality-specific climate data based on a set of 19 bioclimatic variables (Hijmans et al., 2005). To reduce the dimensionality without eliminating bioclimatic variables, we constructed four climatic indexes by applying a PCA on climatic variables following Alvarado-Cárdenas et al. (2013) (Table 1). These four indexes represent annual temperature variation, temperature range or seasonality, variation of precipitation in the most humid season, and variation of precipitation in the driest season. We decided to use the first principal component of each climatic index, as they account for most of the climatic variation in the study area (Table S3). For each specimen we extracted locality-specific climate index data using a geographic information system. We used the climatic index data for each individual as a fixed explanatory variable in the regression models.

**Table 1:** Bioclimatic variables used to construct the climatic indexes.

**Temperature mean variation index**	**Temperature range index (seasonality)**	**Variation of precipitation in humid season**	**Variation of precipitation in the dry season**
**BIO1** = Annual Mean Temperature	**BIO4** = Temperature Seasonality (standard deviation *100)	**BIO13** = Precipitation of Wettest Month	**BIO14** = Precipitation of Driest Month
**BIO5** = Max Temperature of Warmest Month	**BIO7** = Temperature Annual Range (BIO5-BIO6)	**BIO16** = Precipitation of Wettest Quarter	**BIO15** = Precipitation Seasonality (Coefficient of Variation)
**BIO6** = Min Temperature of Coldest Month	**BIO2** = Mean Diurnal Range (Mean of monthly (max temp - min temp))	**BIO12** = Annual Precipitation	**BIO17** = Precipitation of Driest Quarter
**BIO8** = Mean Temperature of Wettest Quarter	**BIO3** = Isothermality (BIO2/BIO7) (* 100)	**BIO18** = Precipitation of Warmest Quarter	**BIO19** = Precipitation of Coldest Quarter
**BIO9** = Mean Temperature of Driest Quarter			
**BIO10** = Mean Temperature of Warmest Quarter			
**BIO11** = Mean Temperature of Coldest Quarter			

**Notes.**

All bioclimatic variables taken from Worldclim 1.4 project (http://www.worldclim.org, Hijmans et al., 2005).

#### Topographic variables

In order to separate the effects of the geographical setting, we used the USGS Digital Elevation Model (altitude, USGS, 2015, https://www.usgs.gov/centers/eros/science/usgs-eros-archive-digital-elevation-global-30-arc-second-elevation-gtopo30?qt-science_center_objects=0#qt-science_center_objects) and aspect as predictor variables in all regression models. To facilitate the use of aspect as a variable that describes topographic orientation, we transformed it using the cosine to express northness and the sine for eastness following Kobelkowsky-Vidrio, Ríos-Muñoz & Navarro-Sigüenza (2014).

### Historical distribution and relatedness data

#### Assemblages of the tyrant flycatchers

**Figure 1:**
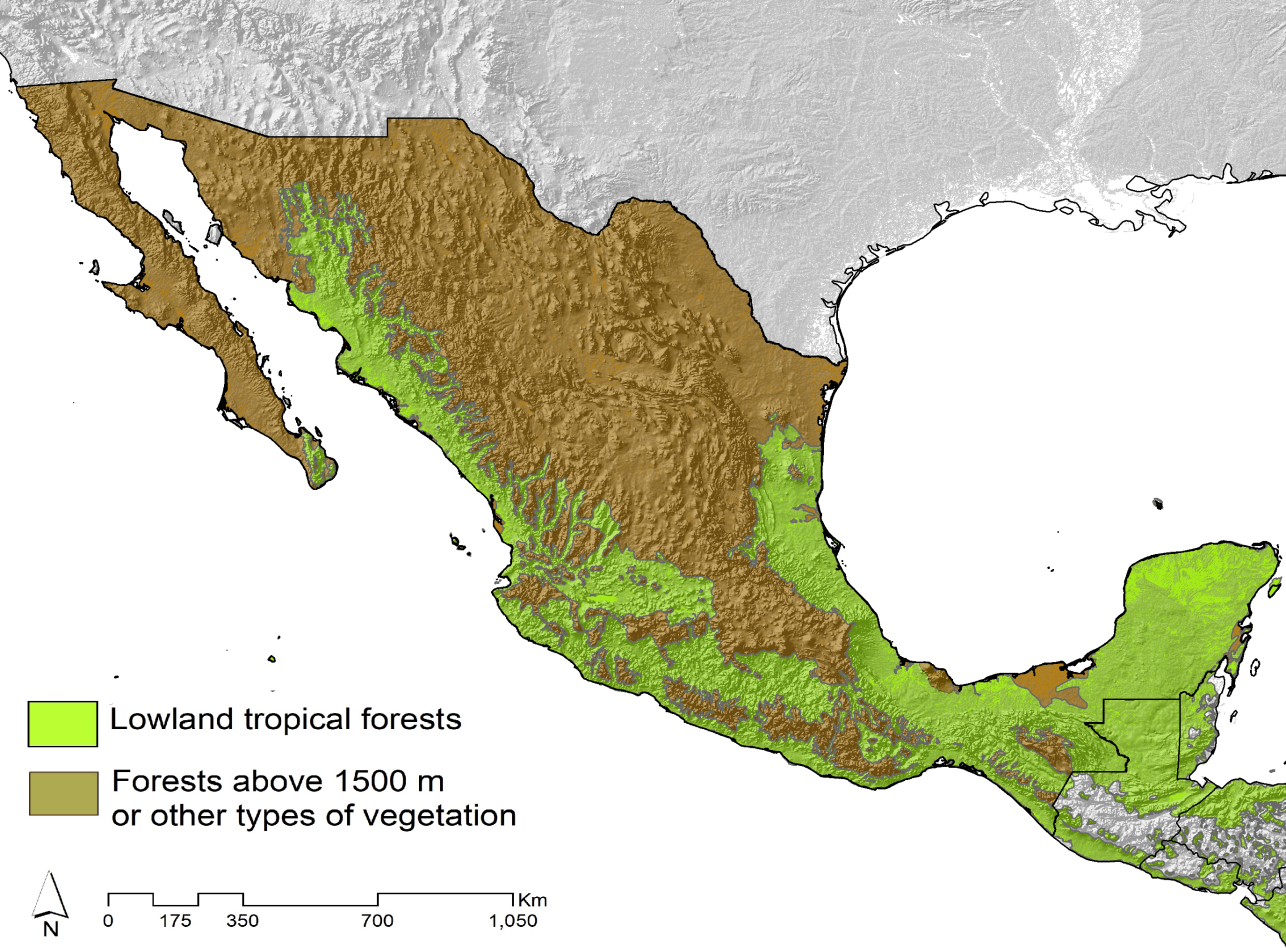
Geographical limits of the three delimited tyrant flycatchers datasets on the basis of the species distributed within Mexico. Areas in green represent the distribution of the lowland tropical dry and humid forests (Assemblage I) and in brown the forests above 1,500 m (highland forests) or other types of vegetation (Assemblage II), the combination of both represents the regional level. Modified from Ríos-Muñoz & Navarro-Sigüenza (2012) and Olguín-Monroy et al. (2013).

In order to discriminate the effects of the evolutionary and historical distribution of the tyrant flycatchers, we divided the data into three separate sets taking into account characteristics of three constructed assemblages of tyrant flycatchers distributed across Mexico. We defined an assemblage as a temporal and spatial arrangement in which species potentially occur and interact; i.e., the pool of species in a geographic area (Halffter & Moreno, 2005, Lessard et al., 2016). We defined two assemblages on the basis of environmental factors delimited by elevation and vegetation type (Fig. 1, Table S1), assemblage I of the lowland forests (species distributed only below 1,500 m) and assemblage II of the highland forests (species present mainly above 1,500 masl) and other types of vegetation, and the Regional level (species distributed in both assemblages, which represent the species distributed in all Mexico). We assigned the species to each assemblage and carried out statistical analysis independently for each data set. We focused on the assemblage I data because Mexican lowland forests are characterized by high levels of species richness, endemism, and habitat specialization, and patterns of biogeographic distribution define them as areas with a particular evolutionary history (Ríos-Muñoz & Navarro-Sigüenza, 2012; Olguín-Monroy et al., 2013). The assemblage II and the Regional level datasets were used to contrast the response of morphological variation to environmental gradients at different spatial scales and community levels. It is known that the influence of different variables on the morphological variation change at different scales of analysis (Lawing et al., 2017).

#### Phylogenetic signal and phylogenetic structure

We reconstructed a phylogenetic tree for the species of Tyrannidae distributed in Mexico using Jetz et al.’s (2012) bird tree with the Hackett et al. (2008) backbone (Fig. S1), in order to calculate the phylogenetic signal of traits and the phylogenetic structure of the localities. The phylogenetic signal was calculated for each morphological variable using the generalized K statistics (Adams, 2014). Phylogenetic signal indicates the tendency of related species to resemble each other more than species drawn at random from the same tree (Blomberg & Garland, 2002). Generalized K statistics tests a null model of evolution of a trait by Brownian motion (drawn at random from the tree), *K* = 1 indicates that trait evolution is consistent with Brownian motion model, while *K* < 1 indicates less similarity in the trait than expected under Brownian motion model, and *K* > 1 indicates greater similarity in the trait than expected under Brownian motion model (Blomberg, Jr & Ives, 2003). Phylogenetic signal tests were conducted using the *geomorph* package (Adams & Otárola-Castillo, 2013) in R version 3.4.1 (R Core Team, 2017).

To determine if the species in a particular area were more closely related than expected by chance, we measured the phylogenetic structure of the Tyrannidae distributed at each locality. To calculate the metric, we used the Net Relatedness Index (NRI, Webb et al., 2002) in the R-package *PhyloMeasures* (Tsirogiannis & Sandel, 2016). Values of NRI greater than zero indicate phylogenetic clustering and values lower than zero indicate phylogenetic evenness or overdispersion. Phylogenetic clustering is found when the co-occurring species of an area are more closely related than expected by chance. Phylogenetic evenness or overdispersion is found when the coexisting species of an area are less related than expected by chance (Webb et al., 2002). To calculate the NRI for each locality, we used the reconstructed phylogenetic hypothesis and we established which species likely co-occur by extracting presence data from distributional hypotheses for Mexican Tyrannidae, generated elsewhere using ecological niche models (Navarro et al., 2004; Dataset S2).

### Statistical analyses

#### The regression models

We evaluated the effects of environmental gradients and phylogenetic structure on morphological variation in the tyrant flycatchers of Mexico using regression models. We constructed trait maps (see below) and obtained our inferences based on the fitting of linear mixed-effects models predicting morphological variation in body size, bill and wing length. We used linear mixed-effects modeling because our data are nested in the sense that samples derive from multiple species, and from each species we have various specimens.

To find the best fitting models for each morphological variable (and assemblage dataset), we followed the protocol recommended by Zuur et al. (2009). In the first step, we started with a model for each morphological variable that contained all the predictor variables and their interaction in the fixed part of the model. There are seven fixed predictor variables (temperature variation index, temperature range or seasonality index, variation of precipitation in humid season, variation of precipitation in the dry season, topographic setting, altitude, and phylogenetic relatedness) and four interactions (relationships between altitude and the temperature and precipitation indexes, Table 2, Table S4 model 1). After obtaining the more complex linear model, we made a new model allowing random intercepts for the nested structure of individuals of a species within a subfamily (Table 2, Table S4 model 2). The randomintercept implies that the basal value of the response is influenced by the nested structure of the data, so measures within a species are more likely to be correlated just because they belong to the same phylogenetic group (Militino, 2010). Next, we allowed random slopes and intercepts for individuals of a species within a subfamily (random intercept), influenced by the phylogenetic relatedness of the communities (random slope, Table 2, Table S4 model 3). Letting the slope to change implies that morphological traits can change between communities in function of how closely related are the species distributed on it. Then, we included the optimal variance structure to the optimal model for the random terms (Table 2, Table S4 model 4). We considered that different variance exist for the observations that have distinct phylogenetic membership. Next, we selected the best fitting model structure for the fixed terms by sequentially adding each predictor variable and their interactions (Table 2) to the optimal random and variance structure model (Table S4 models 5–16). We tested if phylogenetic relatedness influenced morphological variation (Evidence for hypothesis 2, Table S4 model 12) by including it to the best fitting model for the fixed terms. Finally, we included the interaction term between phylogenetic relatedness and the climatic variables that best explained the morphological variation (temperature seasonality, model 17). The interaction between phylogenetic relatedness and temperature seasonality implies that phylogenetic structure modifies the effect of temperature seasonality on the morphological variation within assemblages. The final products of the procedure described were nine best fitting models predicting each morphological variable, at each assemblage, in relation to climatic variables, phylogenetic structure and phylogenetic membership (Table S4, Table 3). We considered the best-fitting model for each variable the one with the highest maximum likelihood (ML), the Akaike information criterion (AIC), and Bayesian informative criterion (BIC, Burnham & Anderson, 2002). We performed all statistical analyses using the *nlme* (Pinheiro, Bates & R-core, 2013) package in R version 3.4.1 (R Core Team, 2017).

**Table 2:** Variables used as fixed terms, interactions and random effects in the regression models for the Mexican tyrant flycatcher.

	**Significance**	**References**
**Morphological variables**	**Response variables**	
**Body mass***(as size proxy)*	Body size is a major influential variable that explains most of the morphological and trait variation within an individual and a species. It is strongly related to their ecology, and also imposes physical constraints to other morphological traits of birds. Body size can predict from the proportion of body parts to the distribution patterns of a species. Its variation has been related to variation in climate and other environmental and phylogenetic factors.	Schmidt-Nielsen (1984), Peters & Peters (1986), Olson et al. (2009), Bonner (2011), Salewski & Watt (2017)
**Wing length**	Wing is considered a major eco-evolutionary module of the birds, that is, a body part identified as an anatomical subregion of the musculoskeletal system that is highly integrated and act as functional unit during locomotion. Wing is related to habitat exploitation and locomotion (bird flight), because of that, wing variation is very physically constrained. For tyrant flycatchers, wing is usually related to the type of habitat that the individual lives in and exploits, as they use a special flights called sallies to catch their prey. Wing shape directly influences evasive movements against predators. Also, the shape and length of the wing are important factors as they directly influence the dispersal ability of birds. Several species of tyrant flycatchers are migratory, so wing length is an important aspect that is directly related to migratory movements.	Hamilton (1961), Fitzpatrick (1980), Fitzpatrick (1981), Fitzpatrick (1985), Miles & Ricklefs (1984), Winkler & Leisler (1992), Gatesy & Dial (1996), Swaddle & Lockwood (1998), Bowlin & Wikelski (2008), Dawideit et al. (2009), Förschler & Barlein (2011)
**Bill variation**	Bill is another major module of the birds, that is, a body part identified as an anatomical subregion of the head that is highly integrated and acts as functional unit during specific processes of the individual, like feeding or communication. For this reason, bill is related to many features of the ecology of the bird, and varies and responds to environmental and evolutionary factors semi-autonomously from other body parts. For tyrant flycatchers, it is most related to their diet breadth and insectivorous feeding habits.	Fitzpatrick (1980), Fitzpatrick (1985), Symonds & Tattersall (2010), Greenberg et al. (2012), Felice & Goswami (2017)
	**Predictor variablesFixed terms**	
**Climatic variables** Mean Temperature Temperature range Variation of precipitation in humid season Variation of precipitation in the dry season	Climatic gradients are part of the environment in which a species occurs. Variables of temperature and precipitation have been related to many functions of organisms and species, as they affect the variation of many morphological traits. For instance body size, distribution range, habitat and diet breadth (niche breadth), reproductive traits, trophic level, and others. In particular, for tyrant flycatchers, mean temperature and range variation could define the suitable areas for occupation and habitat distribution. They also are supposedly major drivers of morphological trait variation. Precipitation seasonality may be related to the distribution of food, as insect abundance within forests and other habitats is correlated with the humid season. Body size and appendage size may be related to climate gradients following the Bergmann’s and Allen’s rules, respectively, as temperature decrease, body size increases but appendage sizes decrease.	Diniz-Filho (2004), Zellweger et al. (2016), O’Donnel & Ignizio (2012), Symonds & Tattersall (2010), Salewski & Watt (2017)
**AltitudeTopographic setting (**northness and eastness)	There is evidence that climatic patterns of precipitation and temperature are affected by altitude. For instance, temperature drops with altitude and precipitation patterns differs with the topographic orientation within a mountainous area (hillshade effect).	Seoane, Bustamante & Diaz-Delgado (2004), Kobelkowsky-Vidrio, Ríos-Muñoz & Navarro-Sigüenza (2014)
**Phylogenetic structure**	Communities are assembled at the local level from regional pools of species, by means of competition and other biotic interactions, and also by the local dispersion or clustering of functional traits. But at the regional scale, the sorting of species, in relation to functional traits can be related to large-scale environmental and climatic gradients. The sorting of individuals at both scales is the result of the combination of the patterns and processes occurring at different scales, and includes a historical component by which the community (or assemblage) is constructed, that is the phylogenetic relatedness of the members of the community. Closely related species can coexist based on the distribution of their functional traits, so the trait composition of the community is predictable because of the sorting of individuals and the history of the community. Then, the phylogenetic structure of a community can potentially explain the distribution of the trait at the community or assemblage scale.	MacArthur & Levins (1967), Webb et al. (2002), Cavender-Bares et al. (2009), Lawing et al. (2017)
**Interaction terms** Altitude x Climatic variables (one interaction with altitude per each climatic index)	As there is clear evidence of the relationship between climate and altitude, we considered that the interaction between the two types of variables) must be considered in the model as a term that might explain morphological variation.	Seoane, Bustamante & Diaz-Delgado (2004)
	**Predictor variablesRandom effect**	
Species of a subfamily at an assemblage influenced by the phylogenetic structure of the communities	Individual’s morphological traits are likely to resemble the morphology of another individual of the same species more closely, simply because they belong to the same phylogenetic group (their shared common ancestry). Measures from individuals of the same species are expected to be correlated; this nested structure potentially violates the statistical assumptions of independence among data, so it has to be considered in the analysis.	Blomberg & Garland, 2002, Blomberg, Jr & Ives (2003), Zuur et al. (2009)
**Variance structure**		
**Phylogenetic membership of species**	Different species groups may have different responses to the fixed terms, thus morphological variables show different dispersion of the data simply because they belong to different groups.	Blomberg & Garland, 2002, Blomberg, Jr & Ives (2003), Zuur et al. (2009)

**Table 3:** Best-fitting models for each morphological trait using mixed-effects model regression.

	**Morphological variable**	**AIC**	**BIC**	**logLIK**	**Model structure**	**Intercept**	**Slope**	***p-*value**
**Regional level**								
	Body size	−167.095	−144.515	90.547	logMass ∼ Temperature seasonality	1.11	0.42	<0.001
	Bill	490.442	503.409	−241.221	logMass ∼ Temperature seasonality	−0.94	0.65	<0.05
	Wing	−431.851	−402.917	224.925	logMass ∼ Temperature seasonality	1.81	0.091	<0.001
**Assemblage I**								
	Body size	−157.429	−128.495	87.714	logMass ∼ Temperature seasonality + phylogenetic relatednessl	1.12	0.56, −0.35	<0.001
	Bill	491.238	504.205	−241.619	logMass ∼ Temperature seasonality + phylogenetic relatedness	−0.94	0.043, 0.03	<0.05
	Wing	−460.550	−444.368	235.275	logMass ∼ Temperature seasonality + phylogenetic relatedness	1.81	−0.002, −0.014	0.45
**Assemblage II**								
	Body size	−178.785	−162.602	94.392	logMass ∼ Temperature seasonality + phylogenetic relatedness	1.11	0.65, 0.60	<0.001
	Bill	513.291	542.226	−247.645	logMass ∼ Temperature seasonality + phylogenetic relatedness	−0.94	0.034, 0.029	0.06
	Wing	−475.085	−462.118	241.542	logMass ∼ Temperature mean variation + phylogenetic relatedness	1.36	−0.004, −0.013	0.141

**Notes.**

logLIK Maximum Likelihood AIC Akaike’s information criterion BIC Bayesian Information Criterion

Assemblage I: Lowland tropical forests. Assemblage II: Highlands above 1,500 masl and other types of vegetation. Regional level the combination of assemblages I and II.

### Mapping the spatial variation of morphological traits

To map the spatial variation of the morphological traits, we extrapolated the best-fitting models into GIS layers. First, we extracted the value of the predictor climatic variable in each pixel (30” resolution) of Mexico within each assemblage. Then, we translated the best-fitting model formula for the climatic index value at each pixel. For instance, if the model was: “Size expected at pixel X = slope*value of climatic index at pixel X + intercept”, we obtained a different value for the morphological variable at each pixel according to the model and the variation of the predictor variable, generating a map of the measurements of the functional traits (Moles et al., 2011). We performed all analyses using the Maptools (Lewin-Koh et al., 2011) package in R version 3.4.1 (R Core Team, 2017). Trait maps were visualized using ArcGIS 10 (ESRI, 2011).

## Results

### Relationship between climatic gradients and morphological variation

Climatic gradients were positively associated with morphological variation of the three measured traits in all three assemblages (Table 3). All best fitting models included at least one climate variable among the fixed terms, specifically, temperature seasonality (temperature range). Temperature appeared to explain variation in morphology at all levels analyzed. At the regional level, for body size, bill and wing length, temperature was related positively and significantly to morphological change, which reflects an increase in the morphological variable values as temperature seasonality increases. The magnitude of the response was higher for body and bill sizes (slopes 0.42 and 0.65, respectively), whereas for wing length was close to zero (slope = 0.091. In other words, wing length does not increase as much as body and bill size with increasing climatic seasonality

For assemblages I and II, the relationship between morphological variables and temperature seasonality was also positive but not significant for some variables. For instance, the regression models for bill size and temperature seasonality, and wing length and temperature seasonality, for assemblage II (highland forests and other types of vegetation) there is no significant relationship between both variables. For assemblage I (lowland forests), the relationship between wing length and temperature sesasonality was not significant either. Only the relationship between body size and temperature seasonality was significantly positive in all assemblages. The relationship between bill variation and temperature seasonality was significant in assemblage I, but the magnitude of the response was less steep than in the assemblage I and the regional level (slope = 0.43).

### Influence of phylogenetic structure on morphological variation

Linear mixed-effects model results indicate that phylogenetic relatedness also helped to explain morphological variation in assemblages I and II, for body size, bill and wing variables (Table 3). In assemblage II (highland forests and other types of vegetation), models for bill and wing were not significant, whereas the model for body size was significant and positively related to phylogenetic relatedness (slope = 0.60). A positive correlation between body size and phylogenetic relatedness means that body size values increase in areas where species that are more closely related co-occur (phylogenetic clustering), and decreases in areas where species that are less closely related co-occur (phylogenetic overdispersion). For assemblage I (lowland forests), phylogenetic relatedness was positively correlated with bill variation, and negatively with body size. The relationship between wing and phylogenetic relatedness was not significant. The results indicate that there is a tendency of decreasing body size when communities become more phylogenetically clustered.

**Figure 2:**
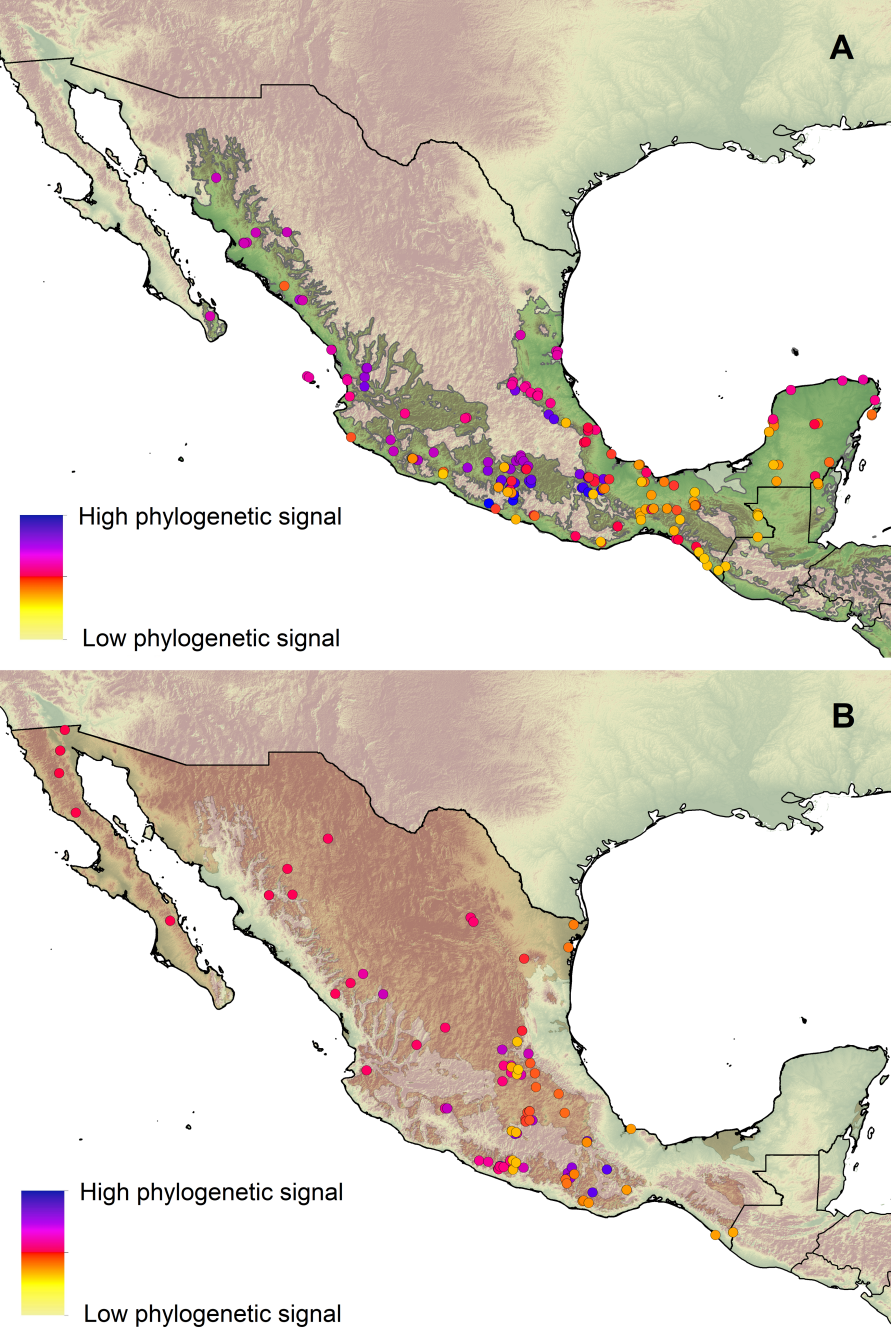
Geographical patterns of phylogenetic signal. (A) Phylogenetic signal at localities of the lowland forests. (B) Phylogenetic signal at localities of the highland forests or other types of vegetation.

Our results indicate that phylogenetic structure exhibits a geographical pattern (Fig. 2). Both assemblages, I and II, comprised areas with phylogenetic overdispersion and phylogenetic clustering (Table S5). Areas of higher phylogenetic clustering appeared to be distributed along the lowland areas of the Balsas Depression, and the highlands of Sierra Madre del Sur (mountain range in the southern Mexico) and Sierra Madre Oriental (mountain range in eastern Mexico). Areas with high phylogenetic overdispersion are mainly distributed in southeastern Mexico (i.e., southeastern Yucatan Peninsula, Tehuantepec Isthmus).

We also measured the phylogenetic signal of the morphological traits, which returned values of *K* = 0.85 for body size, *K* = 0.88 for bill variation, and *K* = 0. 87 for wing length. All values were statistically significant at *α* = 0.05. These values indicate that although the phylogenetic signal for each morphological variable at the species level is lower than 1, values are close to a Brownian motion model, which means that they are slightly less similar than expected due to phylogenetic relatedness.

### Spatial variation of morphological traits in relation to environmental gradients

**Figure 3:**
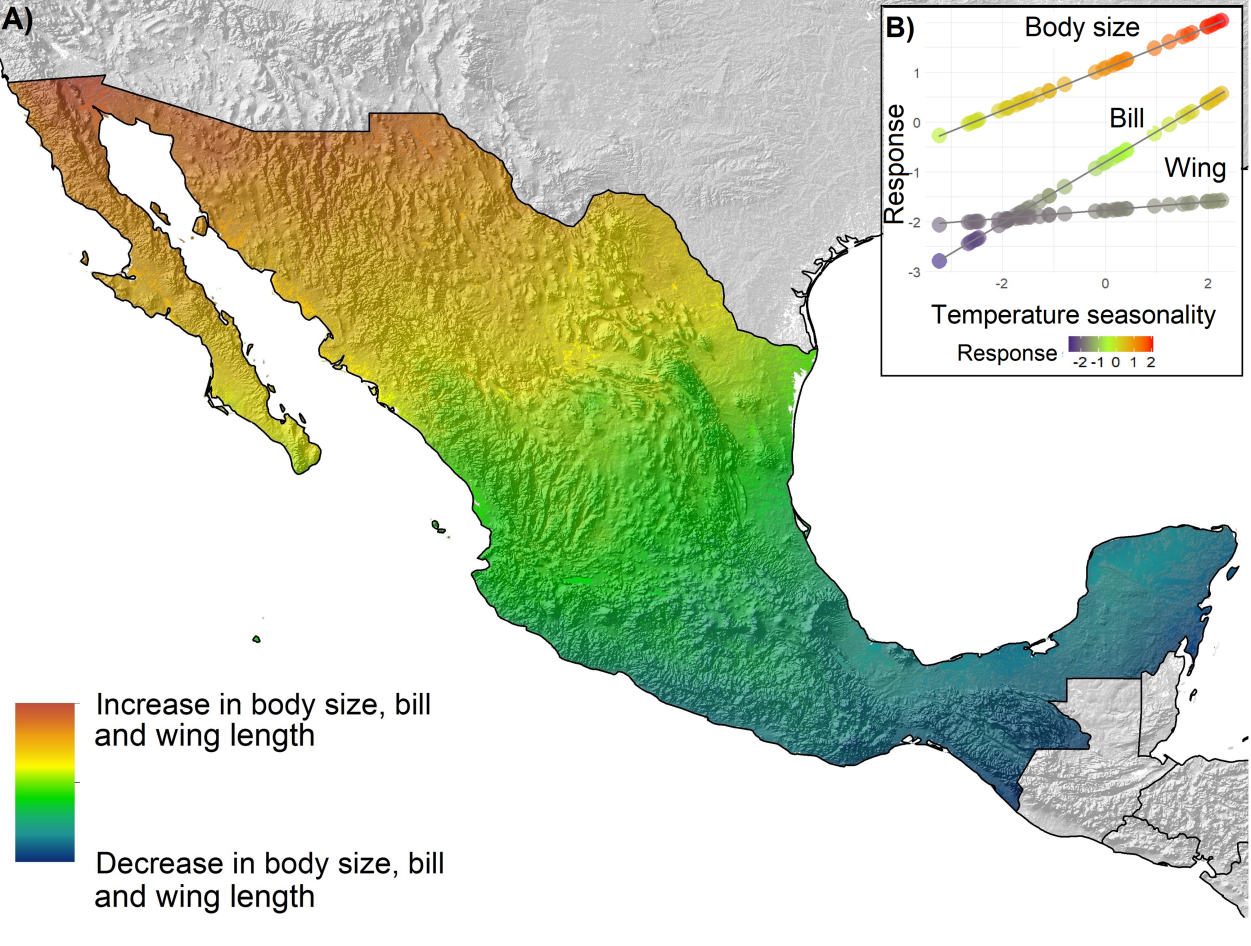
Spatial distribution of morphological variation of body size, bill size and wing length fitted for the regional level by temperature seasonality. (A) Predicted spatial distribution of morphological variation. (B) Scatterplot diagram and regression lines for the predicted response of body size, bill and wing to the increase in temperature seasonality.

**Figure 4:**
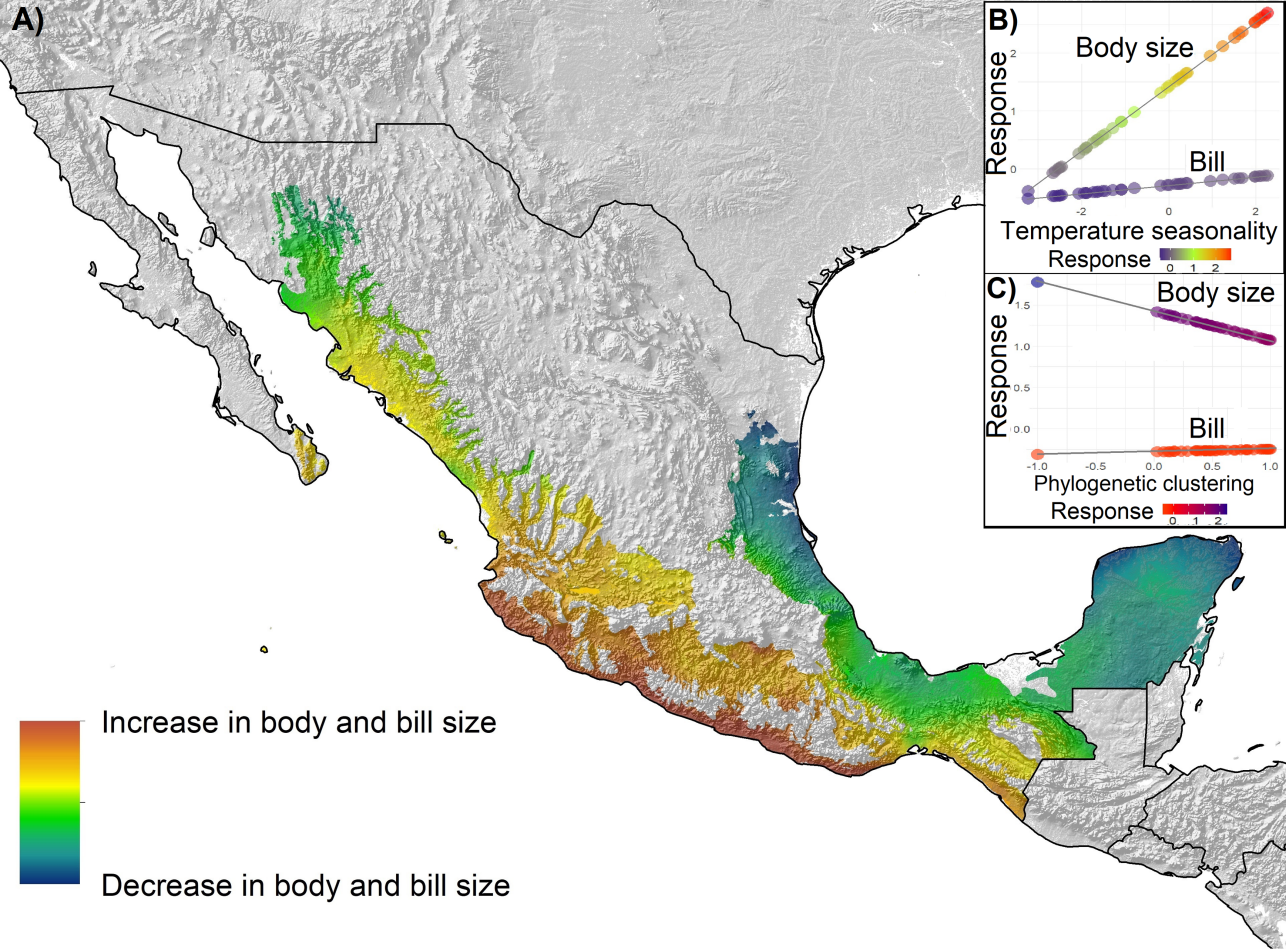
Spatial distribution of morphological variation of body size and bill fitted for Assemblage I by temperature seasonality. (A) Predicted spatial distribution of morphological variation. (B) Scatterplot diagram and regression lines for the predicted response of body size and bill to the increase in temperature seasonality. (C) Scatterplot diagram and regression lines for the predicted response of body size and bill to the increase in phylogenetic clustering.

**Figure 5:**
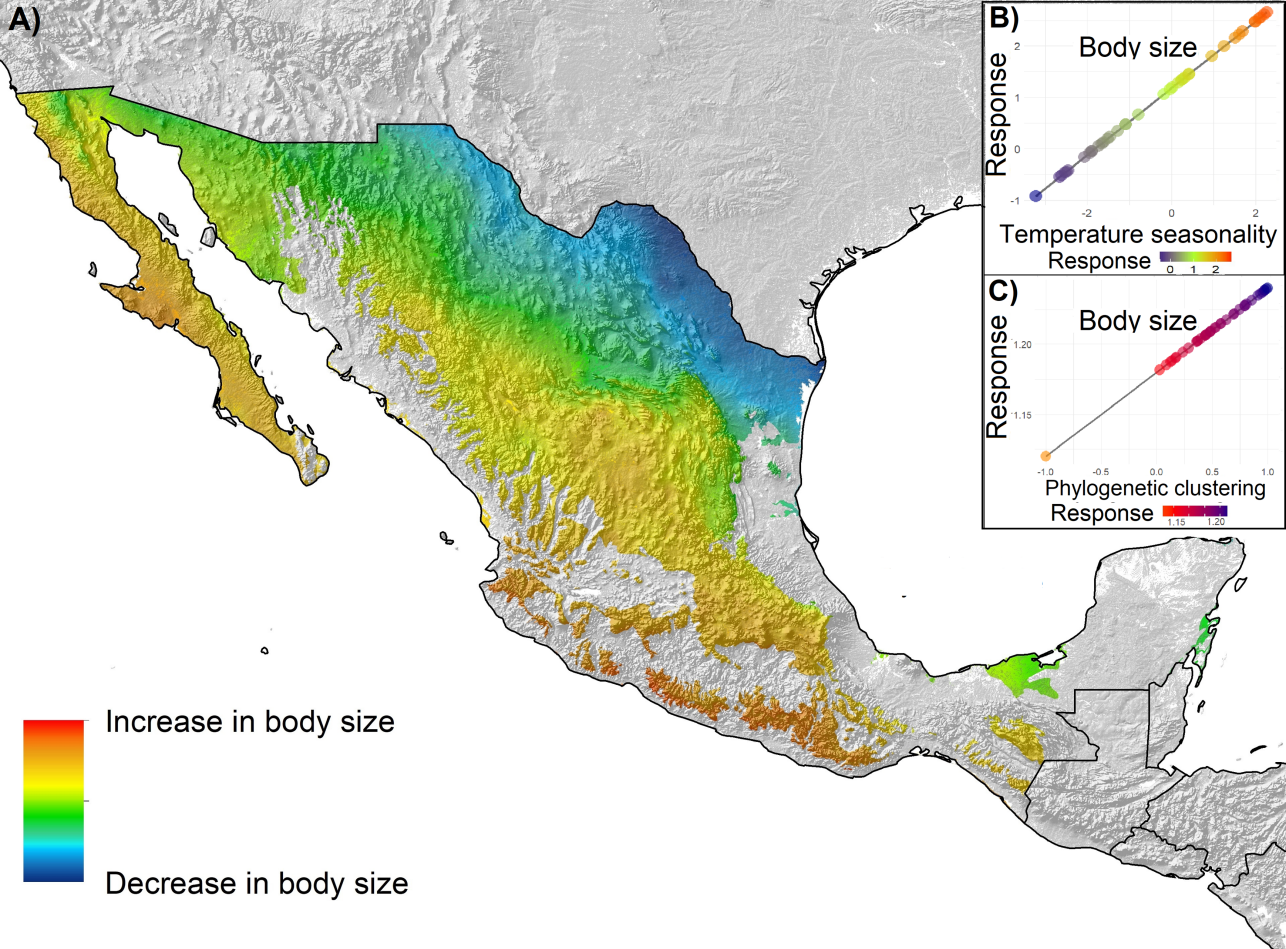
Spatial distribution of morphological variation of of body size fitted for Assemblage II by temperature seasonality. (A) Predicted spatial distribution of morphological variation. (B) Scatterplot diagram and regression lines for the predicted response of body size to the increase in temperature seasonality. (C) Scatterplot diagram and regression lines for the predicted response of body size to the increase in phylogenetic clustering.

Overall, trait variation was explained by temperature gradients and phylogenetic structure at assemblages other than the regional level. Mapping the predictions of the best fitting models (Table 3) yielded different patterns of spatial distribution for morphological variation (Figs. 3–5), across the geography at different scales. Maps represent the gradient of change of the morphological traits with respect to the environmental variable that better explain their variation than other variable. We only mapped the statistically significant models. At the regional level (Fig. 3), for the three morphological variables, morphological trait values increased with increasing latitude. Phylogenetic relatedness does not help to explain morphological variation in the regional level. Assemblage I showed a morphological trait variation from northeast to southwest (Fig. 4), in which body size and bill size increases towards the southwest. In the lowland forests assemblage, bill size increases with increasing phylogenetic relatedness. Conversely, body size increases in areas with low phylogenetic relatedness (overdispersion) and decreases in areas with phylogenetic clustering (Fig. 2A). Geographically this means that phylogenetic relatedness decreases body size in areas where temperature gradients predict an increase in body size, and it increases in body size where temperature gradients predict a decrease. For assemblage II (Fig. 5), we mapped body size and bill variation, which are explained by temperature seasonality. Increases in body size and bill variation were predicted in areas of higher phylogenetic clustering and in southwestern Mexico (Fig. 2B).

## Discussion

Our results suggest that both climatic variables and phylogenetic structure influence the morphological variation of Mexican tyrants, but the influence of the phylogenetic structure varies between different assemblages and morphological traits. When we focused on how climatic gradients explain the variation in morphology, our results suggest that temperature seasonality is the most influential climatic variable, but the magnitude of the influence varies across different assemblages. This variable assumedly represents tolerance limits of species to variation in temperature, likely influencing morphological variation through maintaining habitat use through time (Wiens & Graham, 2005). Our results showed a latitudinal pattern that is consistent with Bergmann’s rule for birds: as temperature increases, body mass is likely to decrease (McNab, 1971). This is a common finding in many studies, because the total surface area of an animal is a proxy for heat dissipation, and predicts that a larger size can be reached in colder climates than in warmer ones, which is linked to the temperature economy of the animal (Salewski & Watt, 2017). Due to the distribution of temperature at the regional level, the latitudinal pattern is likely to show an increase in body size from south to north (Fig. 3), but some studies found exceptions in different regions (e.g., James, 1970).

For assemblages I and II, morphological variation in western Mexico showed a pattern in which the tendency to increase in body size was predicted in direction to both highlands and lowlands of western Mexico (Figs. 4 and 5), which also contain the areas with the highest values of phylogenetic relatedness. A larger body size in less vegetated or highly seasonal areas may be an adaptation to live in these types of isolated environments, and higher phylogenetic relatedness agrees with the fact that western areas have been identified as a complex biogeographical and ecological setting in which a highly endemic and phylogeographically structured bird fauna occurs (e.g., García-Trejo & Navarro-Sigüenza, 2004; Navarro et al., 2004; Ríos-Muñoz & Navarro-Sigüenza, 2012; Arbeláez-Cortés, Milá & Navarro-Sigüenza, 2014). For patterns of morphological variation in the eastern lowlands, like the phylogenetically overdispersed Yucatan Peninsula or the Tehuantepec Isthmus, relatively constant (i.e., less seasonal) temperatures in the east may have influenced the distribution of lineages and the variation of their morphological traits, and consequently the particular phylogenetic community structure in those regions (Martin, MJ & BP, 2018).

The results of several studies support the idea that environmental gradients influence the phylogenetic structure of the communities and therefore, phylogenetic clustering increases with decreasing temperature, meaning that closely related species tend to have a strong phylogenetic signal, and more similar traits and geographic distributions than expected by chance (Helmus et al., 2007; Donoghue, 2008; Graham et al., 2009; Flynn et al., 2011; Tedersoo et al., 2012; Miller, Zanne & Ricklefs, 2013). For instance, Miller, Zanne & Ricklefs (2013) found that the tendency of species to remain in an environmental space similar to that of their ancestors (niche conservatism, Wiens & Graham, 2005) constrains honeyeater assemblages in arid regions, along a gradient of decreasing precipitation. Instead, we found that tyrant’s assemblages became more phylogenetically clustered along a gradient of increasing temperature seasonality, but with low phylogenetic signal. Our findings might reflect that variation in morphological traits of phylogenetically clustered assemblages is more restricted in their climatic ranges. Moreover, on another study, Graham et al. (2009) found that hummingbird communities of the Andean region tend to be phylogenetically clustered at higher elevations and colder areas, and to be overdispersed at lower elevations, whereas in the transition zone between lowlands and highlands there is a species turnover of relatively distant related species that can be associated to the environmental gradient. We found similar results in which phylogenetically clustered communities are found in the western areas (Fig. 2) which includes mountainous ranges above 1,500 masl (southern Sierra Madre Oriental, and the Sierra Madre del Sur), although lowland areas like the Balsas Depression also show high values of phylogenetic clustering.

Phylogenetic clustering at higher elevations supports the idea of environmental filtering, a pattern where similar traits are selected above other variations because they have an advantage within the community and the environment, also allowing the coexistence of close relatives (Webb et al., 2002). Phylogenetic clustering in lowlands like the Balsas Depression supports the idea of the effect of dispersal barriers over community structuring, where communities are phylogenetically similar despite their large differences in species composition, a pattern reflecting the influence biogeographic barriers (Graham et al., 2009) that promote regions with a set of related species with a common and isolated history, like areas of endemism (Harold & Mooi, 1994).

The phylogenetic overdispersion patterns we found could be related to the expectation that competition influences the local trait composition of a community by promoting the filling of the morphological and ecological space exploited (Wainwright & Reilly, 1994); but it could also be associated with the distribution of a lineage along a transition zone, that is, an area where a mixed set of distinct biotic elements overlap (Morrone, 2004). Areas found with higher phylogenetic overdispersion have been recognized by other authors as areas where different biotic elements overlap, e.g parts of the Mexican Transition Zone (Sierra Madre Oriental), Yucatan Peninsula and the limits of the Tehuantepec Isthmus (Morrone, 2006; Morrone, 2014).

Contradictory to the expectations of patterns of phylogenetic structuring, our data show low phylogenetic signal, so traits are less similar than expected due to phylogenetic relatedness. We would have expected a strong phylogenetic signal, as closely related species of a community tend to occupy similar morphological space due to common ancestry, especially in phylogenetically clustered areas. Overdispersion of traits driven by competitive interactions and divergent trait evolution, as well as the taxonomic and spatial scale, may have influenced the results by masking phylogenetic signal patterns at different assemblages (Webb et al., 2002; Cavender-Bares, Keen & Miles, 2006; Lawing et al., 2017). The latter seems to be the case for tyrant flycatchers, as many closely related clades that supposedly have a similar distribution of traits, are concentrated in the same areas of high phylogenetic structure. For example, closely related and morphologically similar *Empidonax* and *Contopus* are concentrated southeastward*,* while another set of closely related *Empidonax* are found concentrated westward (i.e., *E. difficilis, E. occidentalis, E. fulvifrons* and *C. cooperi, C. pertinax and C. sordidulus*). On the other hand, the areas that have more phylogenetically diverse communities (phylogenetic overdispersion) are found in southeastern tropical region, for example the Yucatan Peninsula.

Another pattern revealed by our analyses was defined by the discordant response of variation in body size in relation to temperature seasonality and phylogenetic relatedness (Fig. 4). Our results indicate that body size increases as temperature seasonality increases, but as communities became more phylogenetically clustered, body size decreases, resulting in a trade-off between the influences of temperature seasonality and phylogenetic relatedness over variation in body size. An evolutionary trade-off suggests that the functional trait of body size is limited by the action of another trait of evolutionary and ecological importance, like the relatedness of the species occurring within the community. Trade-offs can occur at different hierarchical levels, and situations can even occur in which the selection on traits of individual organisms is opposed to the selection on an emergent characteristic at the species level (Jablonski, 2007), establishing variation patterns that cannot be fully explained by analyzing a single level. Then, the variation of a characteristic of the individual like body size could be opposed to the selection of a property at the species level (Diniz-Filho, 2004), like the structuring of communities.

## Conclusions

Our analyses demonstrate that the environment has an effect on morphological variation that is mediated by the phylogenetic structure of communities across geography. The use of different environmental variables to elucidate patterns of morphological change in lineages, with distinct levels of phylogenetic signal, and varied patterns of lineage composition across space provides greater explanatory power than only taking into account species richness or abundance, or simply presence/absence distributional data (Olson et al., 2009; Maestri et al., 2016; Lawing et al., 2017). Several authors have noticed that morphological variation is best explained by a varied set of variables, given that the effect of a single climatic variable, most of the time explains variation only at one scale (taxonomic or geographic, James, 1970; Dial, Greene & Irschick, 2008; Olson et al., 2009; Martínez-Monzón et al., 2017). Assessing the distribution of ecomorphological traits of organisms is the best way to predict change over an environmental gradient (Olson et al., 2009; Santos, Cianciaruso & Marco, 2016) and consequently, regression models representing variation of functional traits provide new insights into elucidating the general mechanisms that relate biodiversity across environmental and geographical changes (Violle et al., 2014). A spatial visualization of the predicted response of trait variation in relation to environmental factors can integrate individual and interspecific level responses to evaluate the importance of morphological adaptation in the explanation of broader scale processes. Finally, our results highlight that to allow a better understanding of the spatial distribution patterns of morphological traits, and the processes that promote them in different assemblages, it is necessary to consider the relationship of different ecomorphological traits of the species in conjunction with the phylogenetic composition of the communities.

##  Supplemental Information

10.7717/peerj.6754/supp-1 Table S1 List of species measured and used to reconstruct the phylogenetic hypothesis The table also shows the assemblage were each species is present: (I) Assemblage of the lowland forests. (II) Assemblage of the highland forests and other types of vegetation. All species in the table represent the species of Tyrannidae distributed in Mexico (Ridgely et al., 2005; Berlanga et al., 2008) and were used to reconstruct the phylogenetic hypothesis. Bold X indicates the assemblage in which the species is mainly distributed.

10.7717/peerj.6754/supp-2 Table S2 Bill variation PCA loadings

10.7717/peerj.6754/supp-3 Table S3 Climatic indexes explained variation in PC1

10.7717/peerj.6754/supp-4 Table S4 Mixed-effects model fit by REML for each assemblage and each variable

10.7717/peerj.6754/supp-5 Table S5 Phylogenetic signal at each locality

10.7717/peerj.6754/supp-6 Figure S1 Phylogenetic hypothesis for Tyrannidae distributed in Mexico Phylogenetic tree for the species of Tyrannidae distributed in Mexico was obtained from Jetz et al. (2012) bird tree with the Hackett et al. (2008) backbone.

10.7717/peerj.6754/supp-7 Dataset S1 Tyrannidae database The database contains the measurements taken in biological collections for Tyrannidae specimens, as well as the geographical coordinates of each specimen.

10.7717/peerj.6754/supp-8 Dataset S2 Species distribution models for Tyrannidae occurring in Mexico Maps were taken from a project carried out by Navarro-Sigüenza et al. (unpubl. data). All maps will be openly available at the Geographic information web page of the National Commission for the Study of Biodiversity (CONABIO http://www.conabio.gob.mx/ informacion/gis/)

## Additional Information and Declarations

### Data Availability

The following information was supplied regarding data availability: The raw data measured at biological collections are available along latitude and longitude of each specimen in File S1. These measures were used for all statistical analysis. The structure of the regression models is available in Table S4. The maps used for analysis are available as a Supplemental Dataset File.
