# Supplementary material for: Influence of phylogenetic structure and climate gradients on geographical variation in the morphology of Mexican flycatcher forests assemblages (Aves: Tyrannidae)
Source: PeerJ. 2019 Oct 15;7:e6754. doi: 10.7717/peerj.6754 (PMC6798907; doi:10.7717/peerj.6754)
Supplement: Table S1 — The table also shows the assemblage were each species is present: (I) Assemblage of the lowland forests. (II) Assemblage of the highland forests and other types of vegetation. All species in the table represent the species of Tyrannidae distributed in Mexico (Ridgely et al., 2005; Berlanga et al., 2008) and were used to reconstruct the phylogenetic hypothesis. Bold X indicates the assemblage in which the species is mainly distributed. [file peerj-07-6754-s001.docx]

| **Tyrannidae species** | **Assemblage** | | **Measured** | |
| --- | --- | --- | --- | --- |
|  | **I** | **II** |  |  |
| *Myiopagis viridicata* | **X** | X | X | |
| *Elaenia flavogaster* | **X** | X | X | |
| *Elaenia martinica* | **X** | X | - | |
| *Elaenia frantzii* |  | **X** | X | |
| *Ornithion semiflavum* | **X** |  | X | |
| *Camptostoma imberbe* | **X** | **X** | X | |
| *Zimmerius vilissimus* |  | **X** | - | |
| *Mionectes oleagineus* | **X** | X | X | |
| *Leptopogon amaurocephalus* | **X** |  | X | |
| *Oncostoma cinereigulare* | **X** | X | X | |
| *Poecilotriccus sylvia* | **X** | X | X | |
| *Todirostrum cinereum* | **X** | X | X | |
| *Rhynchocyclus brevirostris* | **X** | X | X | |
| *Tolmomyias sulphurescens* | **X** | X | X | |
| *Platyrinchus cancrominus* | **X** | X | X | |
| *Xenotriccus callizonus* | X | **X** | X | |
| *Xenotriccus mexicanus* | X | **X** | X | |
| *Sayornis phoebe* | X | **X** | X | |
| *Sayornis nigricans* | **X** | **X** | X | |
| *Sayornis saya* | X | **X** | X | |
| *Mitrephanes phaeocercus* | X | **X** | X | |
| *Contopus cooperi* | X | **X** | X | |
| *Contopus pertinax* | X | **X** | X | |
| *Contopus sordidulus* | X | **X** | X | |
| *Contopus virens* | **X** | X | X | |
| *Contopus cinereus* | **X** | X | X | |
| *Empidonax flaviventris* | **X** | X | X | |
| *Empidonax virescens* | **X** | X | X | |
| *Empidonax traillii* | X | **X** | X | |
| *Empidonax alnorum* | **X** | X | X | |
| *Empidonax albigularis* | **X** | **X** | X | |
| *Empidonax minimus* | **X** | **X** | X | |
| *Empidonax hammondii* | **X** | **X** | X | |
| *Empidonax oberholseri* | **X** | **X** | X | |
| *Empidonax wrightii* | **X** | **X** | X | |
| *Empidonax affinis* |  | **X** | X | |
| *Empidonax difficilis* | **X** |  | X | |
| *Empidonax occidentalis* | X | **X** | X |  |
| *Empidonax flavescens* | **X** |  | X |  |
| *Empidonax fulvifrons* | X | **X** | X |  |
| *Legatus leucophaius* | **X** | X | X |  |
| *Pyrocephalus rubinus* | **X** | **X** | X |  |
| *Myiozetetes similis* | **X** |  | X |  |
| *Pitangus sulphuratus* | **X** | X | X |  |
| *Myiodynastes luteiventris* | **X** | X | X |  |
| *Myiodynastes maculatus* | **X** |  | X |  |
| *Megarynchus pitangua* | **X** | X | X |  |
| *Tyrannus melancholicus* | **X** | **X** | X |  |
| *Tyrannus couchii* | **X** | **X** | X |  |
| *Tyrannus vociferans* | **X** | **X** | X |  |
| *Tyrannus crassirostris* | **X** | **X** |  |  |
| *Tyrannus verticalis* | **X** | **X** | X |  |
| *Tyrannus forficatus* | **X** | **X** | X |  |
| *Tyrannus savana* | **X** |  | X |  |
| *Tyrannus tyrannus* | **X** | **X** | X |  |
| *Tyrannus dominicensis* | **X** | X |  |  |
| *Rhytipterna holerythra* | **X** |  | X |  |
| *Myiarchus yucatanensis* | **X** | X | X |  |
| *Myiarchus tuberculifer* | **X** | **X** | X |  |
| *Myiarchus cinerascens* | **X** | **X** | X |  |
| *Myiarchus nuttingi* | **X** | **X** | X |  |
| *Myiarchus crinitus* | **X** | **X** | X |  |
| *Myiarchus tyrannulus* | **X** | X | X |  |
| *Deltarhynchus flammulatus* | **X** |  | X |  |
| *Attila spadiceus* | **X** | X | X |  |
