# Supplementary material for: Influence of phylogenetic structure and climate gradients on geographical variation in the morphology of Mexican flycatcher forests assemblages (Aves: Tyrannidae)
Source: PeerJ. 2019 Oct 15;7:e6754. doi: 10.7717/peerj.6754 (PMC6798907; doi:10.7717/peerj.6754)
Supplement: Table S2 [file peerj-07-6754-s002.docx]

| **Variable** | **PC1** | **PC2** | **PC3** |
| --- | --- | --- | --- |
| **Proportion of variance (%)** | 86.26 | 10.39 | 3.33 |
| **BILL_WIDTH** | 0.552 | 0.819 | -0.158 |
| **BILL_LENGT** | 0.582 | -0.514 | -0.63 |
| **BILL_DEPTH** | 0.597 | -0.255 | 0.761 |
