# Supplementary material for: Influence of phylogenetic structure and climate gradients on geographical variation in the morphology of Mexican flycatcher forests assemblages (Aves: Tyrannidae)
Source: PeerJ. 2019 Oct 15;7:e6754. doi: 10.7717/peerj.6754 (PMC6798907; doi:10.7717/peerj.6754)
Supplement: Table S3 [file peerj-07-6754-s003.docx]

| **Climatic index** | **Proportion of variance (%)** | **PC1 Loadings** |
| --- | --- | --- |
| Temperature variation index | 66.15 | \| BIO1 = Annual Mean Temperature \| 0.147 \| \| --- \| --- \| \| BIO5 = Max Temperature of Warmest Month \| -0.435 \| \| BIO6 = Min Temperature of Coldest Month \| 0.21 \| \| BIO8 = Mean Temperature of Wettest Quarter \| -0.394 \| \| BIO9 = Mean Temperature of Driest Quarter \| 0.117 \| \| BIO10 = Mean Temperature of Warmest Quarter \| -0.452 \| \| BIO11 = Mean Temperature of Coldest Quarter \| 0.208 \| |
| Temperature range index (temperature seasonality) | 78.94 | \| BIO4 = Temperature Seasonality (standard deviation *100) \| 0.29 \| \| --- \| --- \| \| BIO7 = Temperature Annual Range (BIO5-BIO6) \| 0.294 \| \| BIO2 = Mean Diurnal Range (Mean of monthly (max temp - min temp)) \| 0.211 \| \| BIO3 = Isothermality (BIO2/BIO7) (* 100) \| -0.234 \| |
| Variation of precipitation in humid season | 76.16 | \| BIO13 = Precipitation of Wettest Month \| -0.27 \| \| --- \| --- \| \| BIO16 = Precipitation of Wettest Quarter \| -0.272 \| \| BIO12 = Annual Precipitation \| -0.284 \| \| BIO18 = Precipitation of Warmest Quarter \| -0.212 \| |
| Variation of precipitation in the dry season | 67.69 | \| BIO14 = Precipitation of Driest Month \| -0.254 \| \| --- \| --- \| \| BIO15 = Precipitation Seasonality (Coefficient of Variation) \| 0.105 \| \| BIO17 = Precipitation of Driest Quarter \| -2.52E-01 \| \| BIO19 = Precipitation of Coldest Quarter \| -0.178 \| |
