# Supplementary material for: Influence of phylogenetic structure and climate gradients on geographical variation in the morphology of Mexican flycatcher forests assemblages (Aves: Tyrannidae)
Source: PeerJ. 2019 Oct 15;7:e6754. doi: 10.7717/peerj.6754 (PMC6798907; doi:10.7717/peerj.6754)
Supplement: Table S4 [file peerj-07-6754-s004.docx]

|  | | **Model signal** | | | | | |
| --- | --- | --- | --- | --- | --- | --- | --- |
| **1**  **All terms and interactions** | | Response= Temperature variation + Temperature range + Variation of precipitation in humid season + Variation of precipitation in the dry season + Phylogenetic signal + Altitude + Topographic setting + (Altitude x Temperature variation) + (Altitude x Temperature seasonality) + (Altitude x Variation of precipitation in humid season) + (Altitude x Variation of precipitation in dry season) | | | | | |
|  |  | **Assemblage** | **Morphological trait** |  | | | |
|  |  |  |  | **AIC** | **BIC** | **logLIK** | **p-value** |
|  |  | **Regional** | **Body size** | -598.956 | -555.737 | 310.941 | 0.149 |
|  |  |  | **Bill** | 592.38 | 625.773 | -285.802 | 0.06 |
|  |  |  | **Wing length** | -647.035 | -603.683 | 337.001 | 0.07 |
|  |  | **Type I** | **Body size** | -149.703 | -122.189 | 83.409 | 0.086 |
|  |  |  | **Bill** | 559.828 | 574.606 | -275.355 | 0.331 |
|  |  |  | **Wing length** | -690.201 | -703.168 | 341.100 | 0.480 |
|  |  | **Type II** | **Body size** | -184.406 | -167.714 | 97.360 | 0.365 |
|  |  |  | **Bill** | 574.671 | 607.066 | -277.259 | 0.22 |
|  |  |  | **Wing length** | -694.707 | -670.298 | 354.896 | 0.282 |
|  | **Optimal random signal** | | | | | | |
| **2**  **All terms and interactions and a Random intercept** | Response= Model 1 , random= 1~ \| Individuals within Species of a Subfamily at assemblage   \| **Assemblage** \| **Morphological trait** \|  \| \| \| \| \| --- \| --- \| --- \| --- \| --- \| --- \| \| **AIC** \| **BIC** \| **logLIK** \| **p-value** \| \| **Regional** \| **Body size** \| -522.658 \| -484.945 \| 271.332 \| 0.065 \| \| **Bill** \| 548.809 \| 579.746 \| -264.781 \| 0.196 \| \| **Wing length** \| -687.451 \| -641.39181 \| 358.051541 \| 0.03 \| \| **Type I** \| **Body size** \| -161.819 \| -132.078 \| 90.160 \| 0.085 \| \| **Bill** \| 562.242 \| 577.083 \| -276.543 \| 0.049 \| \| **Wing length** \| -689.8547 \| -702.8217 \| 340.9273 \| 0.011 \| \| **Type II** \| **Body size** \| -196.468 \| -178.684 \| 103.728 \| 0.086 \| \| **Bill** \| 545.894 \| 576.667 \| -263.375 \| 0.068 \| \| **Wing length** \| -558.91 \| -543.655 \| 284.160 \| 0.379 \| | | | | | | |
| **3**  **All terms and interactions and a Random intercept and slope** | Response= Model 1, random=1 + phylogenetic signal \| Individuals within Species of a Subfamily at assemblage   \| **Assemblage** \| **Morphological trait** \|  \| \| \| \| \| --- \| --- \| --- \| --- \| --- \| --- \| \| **AIC** \| **BIC** \| **logLIK** \| **p-value** \| \| **Regional** \| **Body size** \| -549.489 \| -509.839 \| 285.261 \| 0.224 \| \| **Bill** \| 505.713 \| 528.293 \| -245.856 \| 0.055 \| \| **Wing length** \| -576.181 \| -537.576 \| 300.097 \| 0.021 \| \| **Type I** \| **Body size** \| -496.468 \| -451.529 \| 262.117 \| 0.23 \| \| **Bill** \| 535.781 \| 549.924 \| -263.528 \| 0.023 \| \| **Wing length** \| -514.604 \| -496.523 \| 262.889 \| 0.061 \| \| **Type II** \| **Body size** \| -564.013 \| -578.901 \| 277.414 \| 0.228 \| \| **Bill** \| 515.068 \| 544.103 \| -248.502 \| 0.064 \| \| **Wing length** \| -534.870 \| -520.272 \| 271.938 \| 0.204 \| | | | | | | |
| **4**  **optimal model for the random terms with different variance signal between observations** | Response= Model 3 + different variance for the observations that have different phylogenetic membership   \| **Assemblage** \| **Morphological trait** \|  \| \| \| \| \| --- \| --- \| --- \| --- \| --- \| --- \| \| **AIC** \| **BIC** \| **logLIK** \| **p-value** \| \| **Regional** \| **Body size** \| -449.224 \| -456.809 \| 213.209 \| 0.131 \| \| **Bill** \| 500.339 \| 528.544 \| -241.396 \| 0.337 \| \| **Wing length** \| -504.111 \| -470.335 \| 262.560 \| 0.043 \| \| **Type I** \| **Body size** \| -188.6595 \| -175.6925 \| 98.32973 \| 0.138 \| \| **Bill** \| 523.33 \| 537.144 \| -257.404 \| 0.019 \| \| **Wing length** \| -527.886 \| -509.338 \| 269.674 \| 0.067 \| \| **Type II** \| **Body size** \| -230.136 \| -209.305 \| 121.503 \| 0.131 \| \| **Bill** \| 536.458 \| 566.699 \| -258.822 \| 0.032 \| \| **Wing length** \| -562.452 \| -547.100 \| 285.961 \| 0.085 \| | | | | | | |
|  | **Best fitting model with no fixed terms** | | | | | | |
| **5**  **Only optimal random signal and optimal variance signal** | Response= Optimal random signal + Optimal variance signal   \| **Assemblage** \| **Morphological trait** \|  \| \| \| \| \| --- \| --- \| --- \| --- \| --- \| --- \| \| **AIC** \| **BIC** \| **logLIK** \| **p-value** \| \| **Regional** \| **Body size** \| 687.352 \| 700.319 \| -339.676 \| 0.06 \| \| **Bill** \| 690.062 \| 703.029 \| -341.031 \| 0.030 \| \| **Wing length** \| -704.806 \| -657.584 \| 367.090 \| 0.135 \| \| **Type I** \| **Body size** \| 684.9457 \| 697.9127 \| -338.4729 \| 0.0297 \| \| **Bill** \| 539.793 \| 554.042 \| -265.501 \| 0.123 \| \| **Wing length** \| -589.241 \| -568.537 \| 301.018 \| 0.46 \| \| **Type II** \| **Body size** \| -654.294 \| -666.681 \| 323.326 \| 0.0242 \| \| **Bill** \| 567.816 \| 599.825 \| -273.951 \| 0.143 \| \| **Wing length** \| -535.876 \| -521.250 \| 272.449 \| 0.511 \| | | | | | | |
| **6**  **Only optimal variance signal** | Response= Optimal variance signal   \| **Assemblage** \| **Morphological trait** \|  \| \| \| \| \| --- \| --- \| --- \| --- \| --- \| --- \| \| **AIC** \| **BIC** \| **logLIK** \| **p-value** \| \| **Regional** \| **Body size** \| -696.111 \| -725.045 \| 339.0557 \| 0.157 \| \| **Bill** \| 695.147 \| 711.3292 \| -342.5735 \| 0.246 \| \| **Wing length** \| 702.6022 \| 655.52788 \| 365.942883 \| 0.111 \| \| **Type I** \| **Body size** \| -690.2011 \| -703.1681 \| 341.1006 \| \| 0.030 \| \| --- \| \| \| **Bill** \| 541.167 \| 555.452 \| -266.177 \| 0.640 \| \| **Wing length** \| -581.602 \| -561.167 \| 297.115 \| 0.228 \| \| **Type II** \| **Body size** \| -695.594 \| -708.763 \| 343.735 \| 0.495 \| \| **Bill** \| 551.068 \| 582.133 \| -265.871 \| 0.129 \| \| **Wing length** \| -527.989 \| -513.578 \| 268.439 \| 0.245 \| | | | | | | |
|  | **Best fitting model signal for the fixed terms**  **Adding terms and interactions to the optimal variance and random signal** | | | | | | |
| **Adding the fixed terms** | **Only climatic gradients explain the morphological variation** | | | | | | |
| **7**  **Temperature and precipitation** | Response= M5 + Variation of precipitation in dry season + Variation of precipitation in humid season + Temperature seasonality+ Temperature variation   \| **Assemblage** \| **Morphological trait** \|  \| \| \| \| \| --- \| --- \| --- \| --- \| --- \| --- \| \| **AIC** \| **BIC** \| **logLIK** \| **p-value** \| \| **Regional** \| **Body size** \| -188.005 \| -174.439 \| 97.600 \| 0.001 \| \| **Bill** \| 494.444 \| 522.317 \| -238.552 \| 0.057 \| \| **Wing length** \| -448.059 \| -425.479 \| 231.029 \| 0.011 \| \| **Type I** \| **Body size** \| -186.807 \| -173.840 \| 97.40394 \| 0.263 \| \| **Bill** \| 492.2699 \| 505.236 \| -242.134 \| 0.030 \| \| **Wing length** \| -497.307 \| -479.833 \| 254.053 \| 0.141 \| \| **Type II** \| **Body size** \| -229.944 \| -209.130 \| 121.402 \| 0.230 \| \| **Bill** \| 536.215 \| 566.442 \| -258.705 \| 0.371 \| \| **Wing length** \| -483.534 \| -470.336 \| 245.838 \| 0.326 \| | | | | | | |
| **8**  **Only precipitation** | Response= M5 + Variation of precipitation in dry season + Variation of precipitation in humid season   \| **Assemblage** \| **Morphological trait** \|  \| \| \| \| \| --- \| --- \| --- \| --- \| --- \| --- \| \| **AIC** \| **BIC** \| **logLIK** \| **p-value** \| \| **Regional** \| **Body size** \| -191.121 \| -177.330 \| 99.218 \| 0.001 \| \| **Bill** \| 499.056 \| 515.238 \| -244.528 \| 0.057 \| \| **Wing length** \| -471.454 \| -458.487 \| 239.727 \| 0.725 \| \| **Type I** \| **Body size** \| -157.081 \| -128.211 \| 87.520 \| 0.001 \| \| **Bill** \| 492.261 \| 505.228 \| -242.130 \| 0.057 \| \| **Wing length** \| -473.773 \| -457.126 \| 242.030 \| 0.461 \| \| **Type II** \| **Body size** \| -191.331 \| -174.012 \| 101.016 \| 0.230 \| \| **Bill** \| 526.729 \| 556.422 \| -254.128 \| 0.15 \| \| **Wing length** \| -480.031 \| -466.929 \| 244.057 \| 0.456 \| | | | | | | |
| **9**  **Only temperature** | Response= M5 + Temperature seasonality + Temperature variation   \| **Assemblage** \| **Morphological trait** \|  \| \| \| \| \| --- \| --- \| --- \| --- \| --- \| --- \| \| **AIC** \| **BIC** \| **logLIK** \| **p-value** \| \| **Regional** \| **Body size** \| -177.411 \| -161.229 \| 93.7059 \| <0.001 \| \| **Bill** \| 492.77 \| 520.548 \| -237.744 \| 0.040 \| \| **Wing length** \| -465.977 \| -449.794 \| 237.988 \| 0.038 \| \| **Type I** \| **Body size** \| -162.118 \| -132.322 \| 90.327 \| <0.001 \| \| **Bill** \| 492.944 \| 505.911 \| -242.472 \| 0.056 \| \| **Wing length** \| -464.882 \| -448.548 \| 237.488 \| 0.423 \| \| **Type II** \| **Body size** \| -187.976 \| -170.961 \| 99.245 \| <0.001 \| \| **Bill** \| 522.952 \| 552.432 \| -252.306 \| 0.412 \| \| **Wing length** \| -478.191 \| -465.139 \| 243.121 \| 0.577 \| | | | | | | |
| **10**  **Only temperature variation** | Response= M5 + Temperature variation   \| **Assemblage** \| **Morphological trait** \|  \| \| \| \| \| --- \| --- \| --- \| --- \| --- \| --- \| \| **AIC** \| **BIC** \| **logLIK** \| **p-value** \| \| **Regional** \| **Body size** \| -186.662 \| -173.695 \| 97.330 \| <0.001 \| \| **Bill** \| 497.581 \| 513.763 \| -243.790 \| 0.030 \| \| **Wing length** \| -476.879 \| -463.912 \| 242.439 \| 0.021 \| \| **Type I** \| **Body size** \| -160.656 \| -131.129 \| 89.512 \| 0.035 \| \| **Bill** \| 496.055 \| 509.149 \| -243.988 \| 0.042 \| \| **Wing length** \| -474.3015 \| -461.3345 \| 241.1508 \| 0.161 \| \| **Type II** \| **Body size** \| -182.308 \| -165.806 \| 96.252 \| 0.04 \| \| **Bill** \| 518.126 \| 547.334 \| -249.978 \| 0.141 \| \| **Wing length** \| -488.244 \| -474.918 \| 248.232 \| 0.260 \| | | | | | | |
| **11**  **Only temperature seasonality** | Response= M5 + Temperature seasonality   \| **Assemblage** \| **Morphological trait** \|  \| \| \| \| \| --- \| --- \| --- \| --- \| --- \| --- \| \| **AIC** \| **BIC** \| **logLIK** \| **p-value** \| \| **Regional** \| **Body size** \| -167.095 \| -144.515 \| 90.547 \| <0.001 \| \| **Bill** \| 490.442 \| 503.409 \| -241.221 \| 0.041 \| \| **Wing length** \| -431.851 \| -402.917 \| 224.925 \| <0.001 \| \| **Type I** \| **Body size** \| -158.821 \| -129.631 \| 88.490 \| <0.001 \| \| **Bill** \| 492.658 \| 520.430 \| -237.690 \| 0.057 \| \| **Wing length** \| -471.7076 \| -458.7406 \| 239.8538 \| 0.141 \| \| **Type II** \| **Body size** \| -183.206 \| -166.623 \| 96.726 \| <0.001 \| \| **Bill** \| 525.88 \| 555.525 \| -253.719 \| 0.638 \| \| **Wing length** \| -484.835 \| -471.602 \| 246.499 \| 0.288 \| | | | | | | |
|  | **Phylogenetic signal influence morphological variation** | | | | | | |
| **12**  **Add to the best fitted model the predictor variable of phylogenetic signal** | Response= M11 + Phylogenetic signal  For Wing length in Type II assemblage:  Response= M10 + Phylogenetic signal   \| **Assemblage** \| **Morphological trait** \|  \| \| \| \| \| --- \| --- \| --- \| --- \| --- \| --- \| \| **AIC** \| **BIC** \| **logLIK** \| **p-value** \| \| **Regional** \| **Body size** \| -187.340 \| -174.373 \| 97.670 \| 0.237 \| \| **Bill** \| 494.262 \| 522.124 \| -238.464 \| 0.055 \| \| **Wing length** \| -473.259 \| -460.292 \| 240.6295 \| 0.298 \| \| **Type I** \| **Body size** \| -157.429 \| -128.495 \| 87.714 \| <0.001 \| \| **Bill** \| 491.238 \| 504.205 \| -241.619 \| 0.04 \| \| **Wing length** \| -460.550 \| -444.368 \| 235.275 \| 0.45 \| \| **Type II** \| **Body size** \| -178.785 \| -162.602 \| 94.392 \| <0.001 \| \| **Bill** \| 513.291 \| 542.226 \| -247.645 \| 0.06 \| \| **Wing length** \| -475.085 \| -462.118 \| 241.542 \| 0.141 \| | | | | | | |
|  | **Altitude and topographic setting influence morphological variation** | | | | | | |
| **13**  **Altitude and topographic setting** | For regional assemblage:  Response= M11 + Altitude + topographic setting  For Type I and Type II assemblages:  Response= M12 + Altitude + topographic setting   \| **Assemblage** \| **Morphological trait** \|  \| \| \| \| \| --- \| --- \| --- \| --- \| --- \| --- \| \| **AIC** \| **BIC** \| **logLIK** \| **p-value** \| \| **Regional** \| **Body size** \| -191.311 \| -178.344 \| 99.655 \| 0.296 \| \| **Bill** \| 494.549 \| 522.427 \| -238.603 \| 0.158 \| \| **Wing length** \| -466.346 \| -450.164 \| 238.173 \| 0.023 \| \| **Type I** \| **Body size** \| -184.167 \| -150.319 \| 102.611 \| 0.140 \| \| **Bill** \| 499.213 \| 527.354 \| -240.853 \| 0.113 \| \| **Wing length** \| -496.791 \| -479.336 \| 253.789 \| 0.807 \| \| **Type II** \| **Body size** \| -209.143 \| -190.212 \| 110.420 \| 0.783 \| \| **Bill** \| 528.655 \| 558.456 \| -255.058 \| 0.514 \| \| **Wing length** \| -502.256 \| -488.547 \| 255.356 \| 0.676 \| | | | | | | |
| **14**  **Only topographic setting** | For regional assemblage:  Response= M11 + topographic setting  For Type I and Type II:  Response= M12 + topographic setting   \| **Assemblage** \| **Morphological trait** \|  \| \| \| \| \| --- \| --- \| --- \| --- \| --- \| --- \| \| **AIC** \| **BIC** \| **logLIK** \| **p-value** \| \| **Regional** \| **Body size** \| -181.971 \| -165.788 \| 95.985 \| 0.27 \| \| **Bill** \| 496.687 \| 512.870 \| -243.343 \| 0.169 \| \| **Wing length** \| -488.005 \| -455.308 \| 254.172 \| 0.034 \| \| **Type I** \| **Body size** \| -191.581 \| -156.370 \| 106.742 \| 0.245 \| \| **Bill** \| 516.822 \| 545.956 \| -249.349 \| 0.158 \| \| **Wing length** \| -482.407 \| -465.457 \| 246.441 \| 0.745 \| \| **Type II** \| **Body size** \| -206.125 \| -187.467 \| 108.827 \| 0.511 \| \| **Bill** \| 521.347 \| 550.736 \| -251.532 \| 0.485 \| \| **Wing length** \| -498.435 \| -484.831 \| 253.414 \| 0.659 \| | | | | | | |
| **15**  **Only Altitude and the interaction** | For regional assemblage:  Response= M11 + Altitude + (Altitude x Temperature seasonality)  For Type I and Type II:  Response= M12 + Altitude + (Altitude x Temperature seasonality)  *For wing length:*  Response= M12 + Altitude + (Altitude x Temperature mean variation)   \| **Assemblage** \| **Morphological trait** \|  \| \| \| \| \| --- \| --- \| --- \| --- \| --- \| --- \| \| **AIC** \| **BIC** \| **logLIK** \| **p-value** \| \| **Regional** \| **Body size** \| -187.976 \| -174.412 \| 97.585 \| 0.326 \| \| **Bill** \| 496.936 \| 524.949 \| -239.754 \| 0.486 \| \| **Wing length** \| -481.578 \| -449.312 \| 250.824 \| 0.041 \| \| **Type I** \| **Body size** \| -192.632 \| -157.228 \| 107.328 \| 0.263 \| \| **Bill** \| 511.65 \| 525.686 \| -251.170 \| 0.304 \| \| **Wing length** \| -489.416 \| -472.220 \| 250.021 \| 0.616 \| \| **Type II** \| **Body size** \| -207.794 \| -188.985 \| 109.708 \| 0.228 \| \| **Bill** \| 547.176 \| 578.021 \| -263.993 \| 0.379 \| \| **Wing length** \| -497.593 \| -484.012 \| 252.985 \| 0.748 \| | | | | | | |
| **16**  **Only altitude** | For regional assemblage:  Response= M11 + Altitude  For Type I and Type II:  Response= M12 + Altitude   \| **Assemblage** \| **Morphological trait** \|  \| \| \| \| \| --- \| --- \| --- \| --- \| --- \| --- \| \| **AIC** \| **BIC** \| **logLIK** \| **p-value** \| \| **Regional** \| **Body size** \| -177.421 \| -164.618 \| 92.105 \| 0.178 \| \| **Bill** \| 492.0478 \| 505.0148 \| -242.0239 \| 0.055 \| \| **Wing length** \| -461.025 \| -430.136 \| 240.119 \| 0.056 \| \| **Type I** \| **Body size** \| -185.723 \| -151.589 \| 103.478 \| 0.146 \| \| **Bill** \| 499.462 \| 512.646 \| -245.664 \| 0.237 \| \| **Wing length** \| -471.645 \| -455.073 \| 240.943 \| 0.467 \| \| **Type II** \| **Body size** \| -188.7035 \| -171.623 \| 99.629 \| 0.119 \| \| **Bill** \| 516.858 \| 545.994 \| -249.366 \| 0.042 \| \| **Wing length** \| -483.234 \| -470.045 \| 245.685 \| 0.356 \| | | | | | | |
|  | **Test for the interaction between best explanatory fixed terms** | | | | | | |
| **17**  **Best fitting signal + interaction between terms** | Response= M12 + Phylogenetic signal * Seasonality  For Wing length in Type II assemblage:  Response= M12 + Phylogenetic signal * Temp. mean   \| **Assemblage** \| **Morphological trait** \|  \| \| \| \| \| --- \| --- \| --- \| --- \| --- \| --- \| \| **AIC** \| **BIC** \| **logLIK** \| **p-value** \| \| **Regional** \| **Body size** \| 490.125 \| 503.041 \| -241.077 \| 0.033 \| \| **Bill** \| 497.995 \| 511.118 \| -244.948 \| 0.002 \| \| **Wing length** \| 496.656 \| 509.744 \| -242.498 \| 0.018 \| \| **Type I** \| **Body size** \| 506.352 \| 528.960 \| -246.167 \| 0.135 \| \| **Bill** \| 515.687 \| 538.244 \| -250.487 \| 0.111 \| \| **Wing length** \| 520.192 \| 543.418 \| -252.895 \| 0.074 \| \| **Type II** \| **Body size** \| 492.4775 \| 508.493 \| -241.289 \| 0.157 \| \| **Bill** \| 407.468 \| 420.719 \| -199.639 \| 0.030 \| \| **Wing length** \| 476.4208 \| 491.914 \| -233.422 \| 0.306 \| | | | | | | |
