# Supplementary material for: Influence of phylogenetic structure and climate gradients on geographical variation in the morphology of Mexican flycatcher forests assemblages (Aves: Tyrannidae)
Source: PeerJ. 2019 Oct 15;7:e6754. doi: 10.7717/peerj.6754 (PMC6798907; doi:10.7717/peerj.6754)
Supplement: Table S5 [file peerj-07-6754-s005.docx]

| **Assemblage** | **Latitude** | **Longitude** | **Phylogenetic structure**  **(NRI)** |
| --- | --- | --- | --- |
| I | 15.5609444 | -93.0168611 | -0.46 |
| I | 15.1 | -92.72 | -0.45 |
| I | 16.6869444 | -93.6683333 | -0.45 |
| I | 15.925 | -96.42 | -0.43 |
| I | 16.677953 | -95.02317 | -0.40 |
| I | 16.303057 | -93.874628 | -0.39 |
| I | 16.6975 | -99.36427 | -0.39 |
| I | 15.338 | -92.828 | -0.35 |
| I | 15.0666667 | -92.0833333 | -0.34 |
| I | 17.0666667 | -94.5833333 | -0.31 |
| I | 16.81 | -90.95 | -0.23 |
| I | 14.9230556 | -92.3372222 | -0.02 |
| I | 15.89417 | -96.4 | -0.39 |
| I | 15.8941694 | -96.3965611 | -0.47 |
| I | 15.7449444 | -93.105 | -0.40 |
| I | 16.0127778 | -93.8052778 | -0.12 |
| I | 22.585 | -105.755 | 0.47 |
| I | 22.5833333 | -105.765 | 0.47 |
| I | 16.44958 | -95.8296382 | -0.38 |
| I | 15.995455 | -93.72381 | -0.40 |
| I | 17.05 | -94.65 | -0.09 |
| I | 16.8166667 | -98.71 | -0.67 |
| I | 17.065 | -100.066667 | -0.57 |
| I | 16.8361667 | -98.74725 | -0.25 |
| I | 17.066819 | -94.1183333 | -0.32 |
| I | 15.99546 | -93.72381 | -0.40 |
| I | 17.18758 | -93.12125 | -0.51 |
| I | 17.5667 | -96.6833 | -0.45 |
| I | 16.84 | -90.98 | -0.42 |
| I | 17.6166667 | -99.5166667 | -0.29 |
| I | 17.6416667 | -93.1566667 | -0.46 |
| I | 17.65 | -99.65 | -0.38 |
| I | 26.66464 | -107.30858 | 0.53 |
| I | 17.75 | -96.3333333 | -0.25 |
| I | 17.7633333 | -96.285 | -0.26 |
| I | 17.816713 | -99.967595 | -0.82 |
| I | 17.8708333 | -93.8833333 | -0.55 |
| I | 17.9 | -88.85911 | -0.33 |
| I | 18 | -95 | -0.37 |
| I | 18 | -94.9 | -0.21 |
| I | 18.005 | -96.6333333 | -0.44 |
| I | 18.0200667 | -90.3200333 | -0.35 |
| I | 18.0277778 | -99.515 | -0.44 |
| I | 18.0875 | -96.1291667 | -0.50 |
| I | 18.1116667 | -96.64 | -0.01 |
| I | 18.1667 | -96.8333 | -0.53 |
| I | 18.2716667 | -101.891667 | -0.65 |
| I | 18.1933333 | -88.98 | -0.15 |
| I | 20 | -89 | -0.37 |
| I | 17.9566667 | -88.885 | -0.33 |
| I | 19.3666667 | -97 | -0.30 |
| I | 21.67 | -106.61 | 0.42 |
| I | 26.6533333 | -108.39 | 0.52 |
| I | 18.3466667 | -101.876944 | -0.20 |
| I | 18.5066667 | -99.77 | -0.40 |
| I | 18.5066667 | -99.7566667 | -0.37 |
| I | 18.5887583 | -95.0979944 | -0.32 |
| I | 18.5927778 | -90.2561111 | -0.28 |
| I | 18.5938889 | -90.4244444 | -0.43 |
| I | 18.6 | -95.0666667 | -0.26 |
| I | 18.68 | -88.4866667 | -0.21 |
| I | 18.8066667 | -102.96 | -0.21 |
| I | 18.8066667 | -102.951667 | -0.28 |
| I | 19.3833333 | -96.9333333 | -0.38 |
| I | 19.5454167 | -105.083861 | -0.63 |
| I | 19.7278833 | -90.5773 | -0.14 |
| I | 19.7699444 | -96.8620833 | -0.27 |
| I | 19.7733611 | -96.8671111 | -0.24 |
| I | 19.8 | -96.6 | -0.02 |
| I | 19.8856306 | -96.8611639 | -0.43 |
| I | 20.0466667 | -97.6233333 | -0.10 |
| I | 20.1761111 | -90.4388889 | 0.24 |
| I | 20.205 | -101.13 | 0.25 |
| I | 22.53682 | -97.96606 | 0.46 |
| I | 20.3716667 | -103.213333 | 0.27 |
| I | 20.73 | -98.1633333 | 0.27 |
| I | 20.8352778 | -86.9005556 | 0.28 |
| I | 20.9608 | -105.1336 | 0.29 |
| I | 20.985 | -98.6066667 | 0.29 |
| I | 20.9856667 | -98.6273333 | 0.29 |
| I | 21.5766667 | -105.225 | 0.41 |
| I | 21.5483333 | -87.315 | 0.41 |
| I | 18.9666667 | -96.05 | -0.07 |
| I | 21.5166667 | -105.216667 | 0.40 |
| I | 21.5095 | -88.0298333 | 0.39 |
| I | 21.4716667 | -99.4630556 | 0.36 |
| I | 21.3611667 | -99.5048333 | 0.35 |
| I | 21.3281 | -99.0063167 | 0.34 |
| I | 21.2733333 | -99.0658333 | 0.32 |
| I | 21.1866667 | -89.8161111 | 0.31 |
| I | 21.0983333 | -98.5916667 | 0.31 |
| I | 21.0933333 | -98.8533333 | 0.31 |
| I | 18.3211111 | -94.8319444 | -0.15 |
| I | 22.516433 | -97.9006 | 0.45 |
| I | 22.40157 | -97.93946 | 0.45 |
| I | 20.225 | -101.078333 | 0.26 |
| I | 18.4369722 | -99.0024444 | -0.43 |
| I | 26.3033333 | -108.698333 | 0.52 |
| I | 16.1147222 | -97.2961111 | -0.24 |
| I | 23.0983333 | -99.2033333 | 0.48 |
| I | 23.7533333 | -109.975 | 0.49 |
| I | 24.3033333 | -106.763333 | 0.50 |
| I | 26.3005 | -108.8225 | 0.52 |
| I | 26.275 | -108.795 | 0.51 |
| I | 21.6261111 | -106.5425 | 0.41 |
| I | 17.7266667 | -99.5616667 | -0.12 |
| I | 17.022226 | -93.78351 | -0.63 |
| I | 28.55 | -109.761667 | 0.54 |
| II | 20.7783333 | -99.5541667 | -0.48 |
| II | 21.7438889 | -99.5905556 | -0.48 |
| II | 20.6066667 | -99.6083333 | -0.48 |
| II | 17.55 | -99.6333333 | -0.48 |
| II | 18.6 | -99.6333333 | -0.47 |
| II | 17.3066667 | -99.74 | -0.45 |
| II | 17.6263 | -99.748522 | -0.41 |
| II | 18.65 | -99.7833333 | -0.40 |
| II | 18.65 | -99.7833333 | -0.40 |
| II | 15.1316667 | -92.11 | -0.39 |
| II | 16.51666 | -92.11 | -0.39 |
| II | 15.0416667 | -92.725 | -0.30 |
| II | 18.7166667 | -95.65 | -0.29 |
| II | 16.6066667 | -95.8 | -0.27 |
| II | 16.155 | -97.1166667 | -0.27 |
| II | 16.2153 | -92.725 | -0.27 |
| II | 16.235 | -97.29 | -0.69 |
| II | 16.2153 | -97.3266 | -0.68 |
| II | 17.1516667 | -97.6183333 | -0.67 |
| II | 25.7983333 | -97.635 | -0.67 |
| II | 25.01 | -97.82 | -0.67 |
| II | 16.8283333 | -97.88 | -0.66 |
| II | 16.9667 | -97.9167 | -0.57 |
| II | 19.2716667 | -97.9466667 | -0.57 |
| II | 19.9330556 | -98.1566667 | -0.56 |
| II | 20.1666667 | -98.9483333 | -0.56 |
| II | 20.645 | -98.9783333 | -0.55 |
| II | 20.9983333 | -99.145 | -0.48 |
| II | 19.0233333 | -99.16 | -0.47 |
| II | 19.3333 | -99.1667 | -0.47 |
| II | 19.335 | -99.1716667 | -0.46 |
| II | 19.295 | -99.24 | -0.46 |
| II | 18.9716667 | -99.2933333 | -0.45 |
| II | 18.2516667 | -97.1833333 | -0.36 |
| II | 18.9716667 | -99.2933333 | -0.35 |
| II | 19.0483333 | -99.3083333 | -0.35 |
| II | 24.6166667 | -99.35 | -0.35 |
| II | 22.1216667 | -99.425 | -0.34 |
| II | 23.4027778 | -105.901667 | -0.27 |
| II | 31.05 | -115.466667 | 0.12 |
| II | 31.8286111 | -115.431667 | 0.13 |
| II | 32.545 | -115.271667 | 0.24 |
| II | 29.67819 | -114.89248 | 0.24 |
| II | 25.9311111 | -111.620833 | 0.24 |
| II | 26.82 | -108.2 | 0.25 |
| II | 27.73255 | -107.55798 | 0.25 |
| II | 26.8380556 | -107.378611 | 0.27 |
| II | 28.7816667 | -106.151667 | 0.27 |
| II | 23.7721944 | -105.368889 | 0.35 |
| II | 20.7490556 | -104.816944 | 0.35 |
| II | 21.632111 | -103.075786 | 0.35 |
| II | 22.229398 | -102.089726 | 0.35 |
| II | 26.04294 | -101.208805 | 0.39 |
| II | 26.04294 | -101.208805 | 0.39 |
| II | 25.895026 | -101.10721 | 0.39 |
| II | 17.3333333 | -100.25 | 0.40 |
| II | 17.3216667 | -100.241667 | 0.40 |
| II | 17.4166667 | -100.2 | 0.40 |
| II | 20.3736111 | -100.103333 | 0.41 |
| II | 20.9125 | -100.065833 | 0.46 |
| II | 17.3958333 | -100.064722 | 0.46 |
| II | 17.6158333 | -99.8386111 | 0.47 |
| II | 20.6718333 | -99.8071667 | 0.47 |
| II | 17.58668 | -99.83707 | 0.57 |
| II | 20.8610333 | -99.7871167 | 0.68 |
