# Supplementary figures and images for: Influence of phylogenetic structure and climate gradients on geographical variation in the morphology of Mexican flycatcher forests assemblages (Aves: Tyrannidae)
Source: PeerJ. 2019 Oct 15;7:e6754. doi: 10.7717/peerj.6754 (PMC6798907; doi:10.7717/peerj.6754)

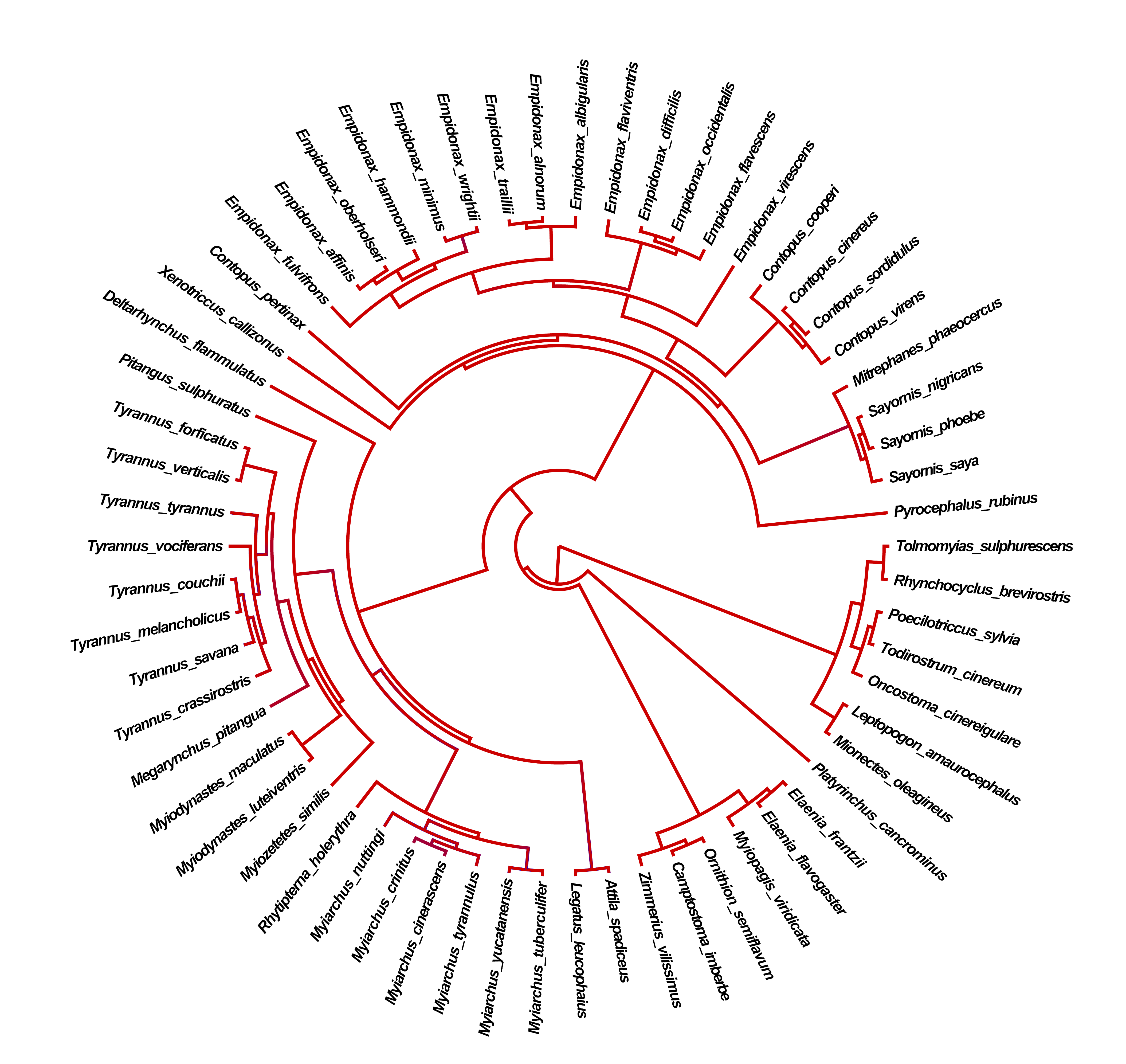

Supplement: Figure S1 — Phylogenetic tree for the species of Tyrannidae distributed in Mexico was obtained from Jetz et al. (2012) bird tree with the Hackett et al. (2008) backbone. [file peerj-07-6754-s006.png]
